# The visual system of a nocturnal long-distance migrant, the Australian Bogong moth

**DOI:** 10.1007/s00359-025-01786-x

**Published:** 2025-12-18

**Authors:** Kristina Brauburger, Willi Ribi, Emelie Svensson, Paul Clémençon, Sho Yee Carisa Goh, Marjorie A. Liénard, Eric Warrant, Stanley Heinze

**Affiliations:** 1 Lund Vision Group, Department of Biology, Lund University, Lund, Sweden; 2 NanoLund, Lund University, Lund, Sweden; 3 GIGA-R, Laboratory of Molecular Biology of Sensory Systems, University of Liège, Liège, Belgium; 4 Australian National University, Canberra, Australia; 5 Département de biologie, Master biologie, École normale supérieure de Lyon, Lyon, France

**Keywords:** Lepidoptera, Noctuid, Migration, Ocelli, Opsins

## Abstract

The Australian Bogong moth (*Agrotis infusa*) is a small noctuid moth that undertakes annual, nocturnal migrations of up to 1000 km to escape the summer heat of its breeding grounds. The moths travel to cool alpine caves, where they enter a dormant state (aestivation) before returning to reproduce and die. During migration, their brains integrate magnetic and visual cues to guide their flight direction. While the Bogong moth’s neurobiology is increasingly understood, its visual system has remained unexplored. Here, we describe the morphology, ultrastructure, optics, and visual opsins of the Bogong moth’s compound eyes and ocelli. Using light and electron microscopy, micro-computed tomography, in situ hybridization, and spectral absorbance measurements, we show that the compound eyes are typical superposition eyes with a tiered rhabdom, similar to other noctuid moths. The ocelli are small but structurally complex, featuring a two-tiered retina with spectrally distinct receptor cells and a lens forming a focused image on the ocellar retina. At the molecular level, the Bogong moth expresses three canonical opsins (UV, blue, and long-wavelength) and an additional red-shifted long-wavelength opsin, suggesting enhanced sensitivity to long-wavelength light. These opsins exhibit distinct expression patterns across the compound eyes, indicating functionally distinct dorsal and ventral eye hemispheres. Overall, the Bogong moth’s visual system displays multiple adaptations to nocturnal vision. These features, likely shared across noctuid moths, may have contributed to the evolution of the exceptional navigational abilities during long-distance migrations in dim light that define the Bogong moth, but which are also widespread across noctuid moths.

**Supplementary Information:**

The online version contains supplementary material available at 10.1007/s00359-025-01786-x.

## Introduction

**Fig. 1:**
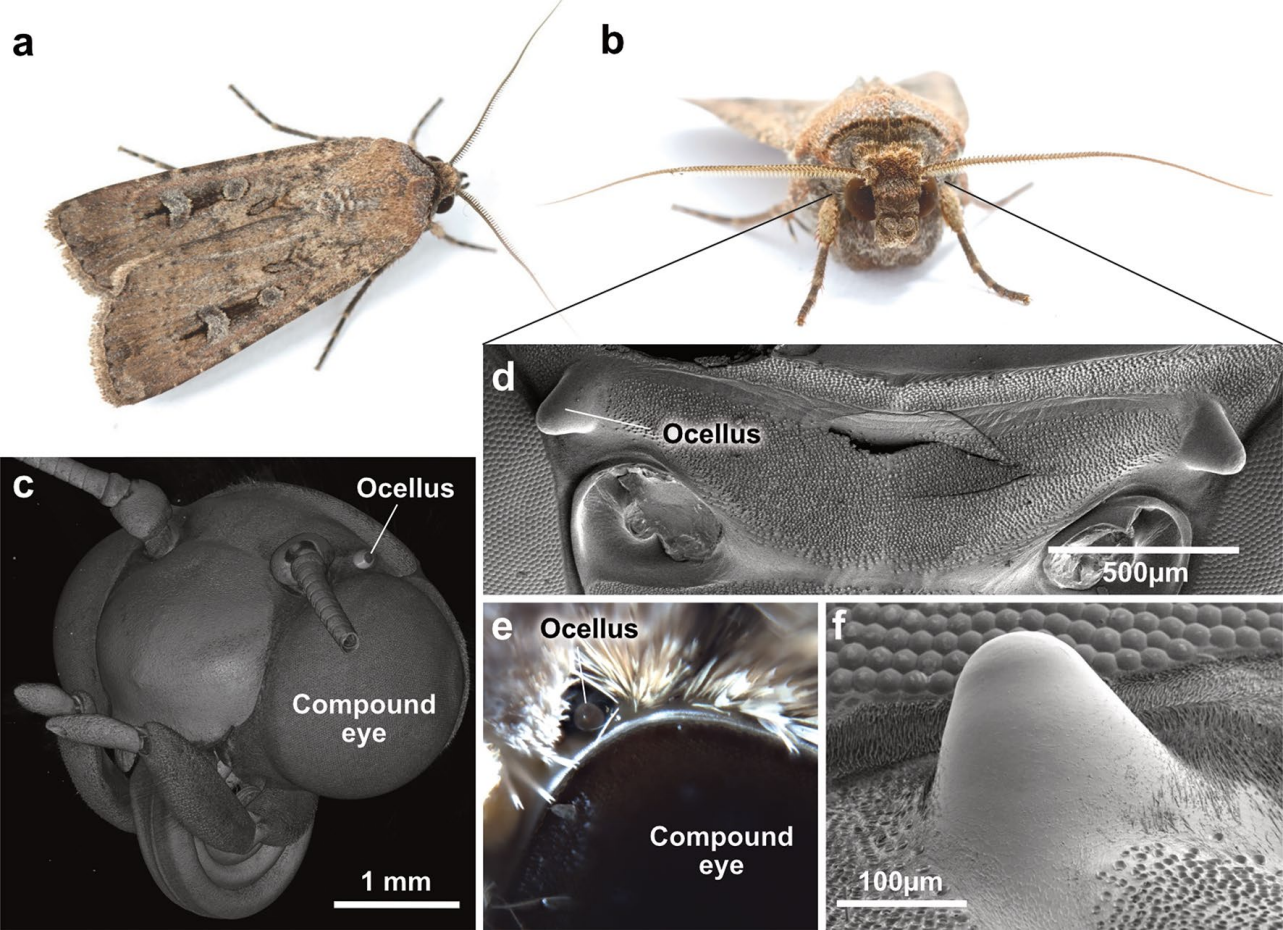
External morphology of the Bogong moth visual system. **a, b** Images of a male Bogong moth. Photographs: Ajay Narendra, Macquarie University. **c** $\upmu$CT based volume rendering of a Bogong moth head, showing relative positions of left compound eye and ocellus; scales not rendered. **d** Scanning electron micrograph (SEM) of dorsal head, with scales removed, highlighting position of ocelli on the head surface. **e** Photograph of ocellus and dorsal compound eye, showing ocellar lens peeking out from in between scales. **f** SEM of the dome shaped ocellus, with the surrounding scales removed

The Bogong moth (*Agrotis infusa*, Fig. 1a, b) is the most iconic lepidopteran species in Australia (Warrant et al. 2016). It owes this fame in part to its ability to disrupt Australian politics by occasionally amassing in the millions in the capital Canberra, sometimes clogging the ventilation shafts of the Parliament building with their tightly packed bodies. But this species has a much deeper relation to Australian culture, reaching back thousands of years. Millennia before any European settlers arrived, the traditional owners of the land gathered each summer in the alpine regions near Canberra to feast on the billions of Bogong moths arriving in synchrony in the mountains (Stephenson et al. 2020). These annual gatherings were reported by explorers in the early 1800s, reports that are backed up both by the oral history of the Aboriginal tribes as well as archaeological records from the mountains (Flood 1980). This way, the Bogong moths brought together many tribes, providing opportunities to foster inter-tribal relationships, and thus served as a lubricant to harmonize Aboriginal relations on a sub-continental scale for thousands of years. In the process, the moth became an integral part of Aboriginal art and cultural identity.

Like the tribes gathering from many directions in the small region occupied by the Australian alps, Bogong moths also arrive from many directions to ultimately aggregate in a relatively small number of inconspicuous alpine caves, formed by the cracks and crevices in between massive boulders piled up near the mountain tops. There, the moths densely tile the cave walls, reaching densities of up to 17,000 moths per square meter (Common 1952). Exploiting the constant cool and humid climate inside the sheltered caves, the moths enter a dormant state called aestivation and thereby escape the deadly summer heat in their breeding grounds (Lownds et al. 2023), located about a thousand kilometers north or west. Once the summer nears its end, the moths once more take flight and return to these fertile regions to mate, oviposit and die. In the following spring, the next adult generation embarks on the same journey towards the life-saving alpine caves (Warrant et al. 2016; Common 1954).

This ability to migrate over very long distances and locate a specific set of mountain caves is remarkable, not only because it is carried out by a 3 cm long animal that weighs only a few hundred milligrams, but because each individual does this only once in its life. Hence, each moth migrating to the caves is always on its first journey, relying exclusively on its inherited sense of direction along the way. The sensory cues encountered during their nocturnal journey allow them to identify the correct compass heading, and locate their target cave - possibly the same cave that was occupied by their ancestors. While close-range identification of their ultimate destination likely relies on olfactory cues (Rosberg et al., in preparation), sensory guidance along the long-distance part of the moth’s journey is achieved by using a combination of magnetic and visual information (Dreyer et al. 2018b), most prominently the starry night sky. In fact, unlike for birds and humans, in which guidance by stars is well documented (Emlen 1975; Mouritsen and Larsen 2001; Lewis 1994), the Bogong moth is the only invertebrate known to date to obtain compass information from the starry night sky to guide its long-distance navigation (Dreyer et al. 2025).

Alongside other visual navigation cues - such as polarized skylight, spectral and intensity gradients across the sky, or visual landmarks - starry sky cues are collected through the visual system and decoded by higher-order brain centers. Yet, while the brain of the Bogong moth is relatively well described (Adden et al. 2020), little is known about the visual system of this species.

The Bogong moth is a nocturnal member of the Noctuidae, one of the largest lepidopteran families (Zahiri et al. 2013). Noctuids typically possess large superposition compound eyes (Fig. 1c), an eye design that significantly increases optical sensitivity in dim light (Warrant 2004; Warrant et al. 2003). As the primary visual organs of these insects, they enable high-resolution image formation, motion, color and object detection (Warrant 2017). Noctuid moths also possess one pair of lateral ocelli (Dow and Eaton 1976). These small, simple lens eyes (Fig. 1d–f) are found in most insects and vary in number, shape, and field of view (Mizunami 1995; Ribi and Zeil 2018; Taylor et al. 2016; Ribi and Zeil 2017). While poorly understood in most species, ocelli of a few insects are well studied. To the best of our knowledge, these include the fruit fly, several bee species, a locust (*Locusta migratoria*), a cockroach (*Periplaneta americana*), and two dragonfly species (*Hemicordulia tau* and *Aeshna mixta*), where ocelli contribute to a diverse range of functions, such as flight stabilization, horizon alignment, optomotor responses, polarized light detection, luminance signaling, and phototactic responses, possibly even enabling color constancy (Berry et al. 2007a; Stange et al. 2002; Honkanen et al. 2017; Garcia et al. 2017; Araújo et al. 2024). These functions complement the functions of the high-resolution, image-forming compound eyes.

Irrespective of which type of eye is used to detect light stimuli, the visual pigments that absorb photons are opsin-chromophore complexes. These molecules are located in the microvillous membranes of the photoreceptor cells and are tuned to maximally absorb light of a specific peak wavelength, resulting in the ability to discriminate colors. Most moths, including noctuids, possess a standard repertoire of three opsins, namely long (LW), short (SW), and ultraviolet (UV) wavelength-sensitive opsins. Yet, LW opsin duplications have occurred relatively frequently in the genomes of diverse moth lineages (Mulhair et al. 2023). This includes the superfamily Noctuoidea, which has retained an ancient LW2 opsin retrogene copy in addition to its standard three-opsin repertoire (UV, SW, LW1) (Xu et al. 2016; Mulhair et al. 2023). This LW2 retrogene opsin is expressed in larvae of *Helicoverpa armigera*, but it is unknown whether LW2 is a functional opsin in the adult visual system of noctuid moths.

Whereas visual opsins enable light detection in compound eyes and ocelli, both eye types do not always share identical opsins. Ocellus-specific long-wavelength opsins have been identified in honey bees (Spaethe and Briscoe 2004; Velarde et al. 2005), crickets (Henze et al. 2012), dragonflies (Futahashi et al. 2015) and flies (blue-violet Rh2) (Pollock and Benzer 1988; Guignard et al. 2022). These often occur together with a UV opsin and, compared to the compound eyes, form ocellar photopigments with distinct UV-green spectral specializations.

This overall versatility of visual systems across insects, both at the morphological and molecular level, suggests that the eyes are subject to vigorous natural selection. Thus, species-specific adaptations in the architecture or molecular tool kit of the eyes might be an effective way to evolve distinct life history traits, such as a migratory lifestyle.

In this study, we therefore set out to comprehensively characterize both the compound eyes and the ocelli of the Bogong moth, and examine if they harbor any specializations that could contribute to the Bogong moth’s migratory ability. First, we obtained detailed information about the anatomical layout of both structures using light and electron microscopy and describe the optical properties of the ocelli. We then characterized the opsin repertoire and examined the expression pattern of each opsin in both the ocellar and compound eye retinae. Finally, we determined the opsin absorption spectra by reconstituting active opsin-retinal complexes *in vitro*, yielding data that allow us to estimate the Bogong moth’s visual capabilities in unprecedented detail.

## Methods

### Animals

Animals used throughout this study were Bogong moths (*Agrotis infusa*) of both sexes, sampled during their aestivating state, unless explicitly stated otherwise. Moths were collected while aestivating in alpine caves, or using light traps during migration, both in Kosciuszko National Park, New South Wales, Australia, between 2015 and 2025. For migratory moths, a state resembling natural aestivation was induced by crowded conditions in holding boxes after collection. Moths were either used locally and processed at the Australian National University (ANU), Canberra, or transported to Sweden in cooled plastic containers. In Lund, moths were maintained in an aestivating state in a temperature-controlled incubator (I-30VL, Percival/CLF Plant Climatics, Wertingen, Germany) in cave-like conditions (16 h dim light at 10$^{\circ }\,\hbox {C}$, 8 h dark at 6$^{\circ }\,\hbox {C}$) for up to six months. The moths had free access to food solution at all times (2% sugar, 2% honey, and 0.2% ascorbic acid in water). Specific animal conditions and handling procedures are indicated for each experimental step below.

### Histology

**Fig. 2:**
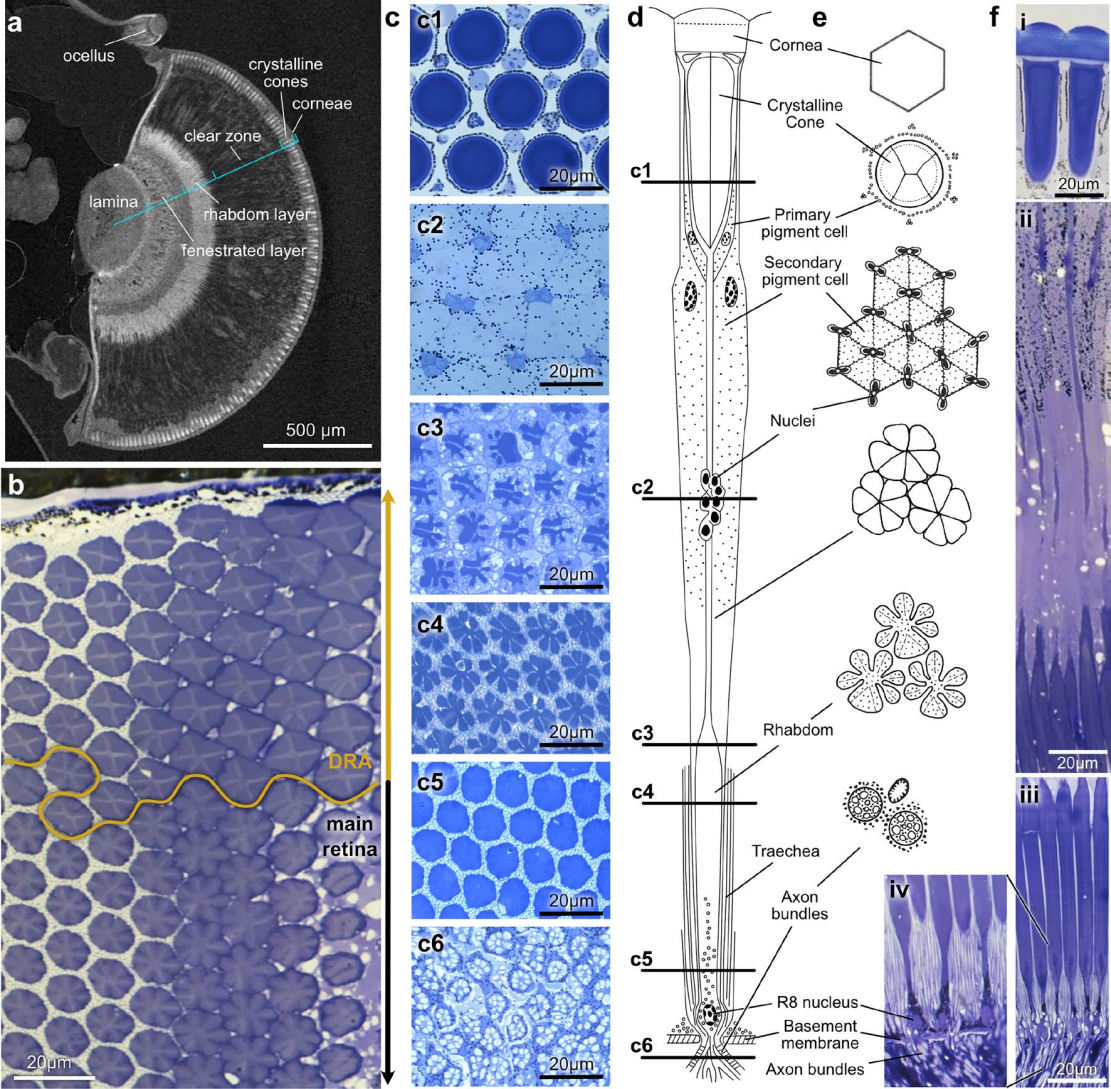
Morphology of the Bogong moth compound eye. **a** Cross-sectional view of the eye obtained via $\upmu$CT, displaying the major internal optical and neural structures of the eye. **b** Light microscopical cross-section through the dorsal eye, illustrating the difference in rhabdom structure between the ommatidia of the dorsal rim area (DRA) and the main retina. **c** Light microscopy sections of individual ommatidia at different levels between the eye surface and the basement membrane. Shown are the crystalline cone (c1), the secondary pigment cells with retinula cell nuclei (c2), three successive levels of the rhabdom (c3–c5), and the photoreceptor axon bundles just beneath the basement membrane. **d** Schematic diagram of a typical light-adapted ommatidium, indicating the cross section levels shown in c and all major cell types contributing to the eye. **e** Schematic drawings of cross sections, highlighting key structures shown in images in c. **f** Longitudinal sections through the Bogong moth retina, showing the cornea and crystalline cone alongside the primary pigment cells (i), the clear zone, including secondary pigment cells, retinula cell nuclei, rhabdom threads and the distal tip of the widened rhabdom (ii), the wide portion of the rhabdom (iii), and the proximal end of the rhabdom, including the nuclei of the R8 retinula cell, the traecheal sheath that surrounds each rhabdom, the basement membrane and the receptor cell axon bundles (iv)

**Fig. 3:**
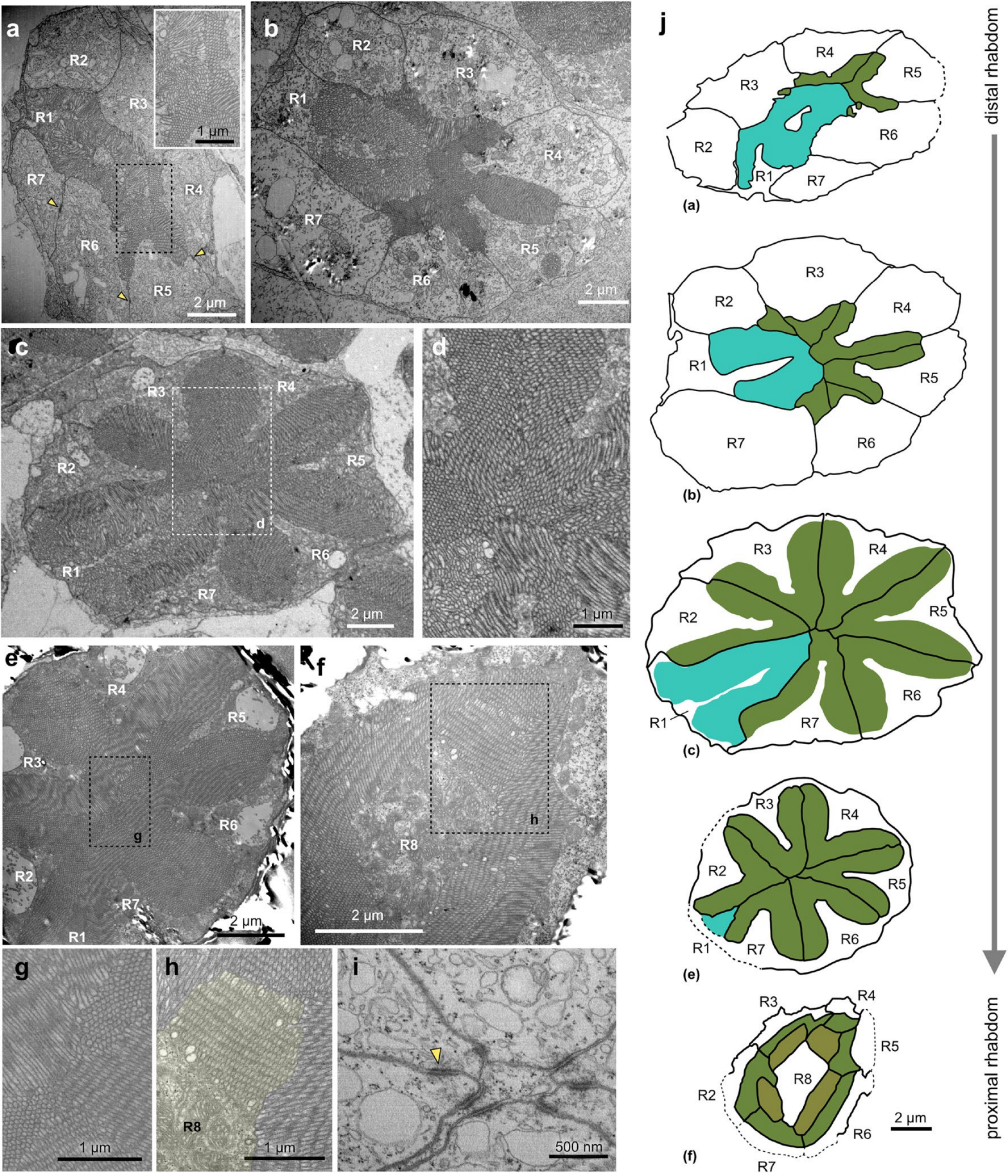
Ultrastructure of the compound eye rhabdoms. **a–h** Transmission electron micrographs (TEM) of cross sections through the rhabdom, progressing from distal to proximal. **a–b** In the distal rhabdom, seven cell bodies are visible, with prominent microvillar contributions from R1 and gradually increasing contributions from R2-R7. Arrowheads: desmosomes linking adjacent receptor cells. The insert highlights the comparably irregular microvilli orientation in R1. **c–d** Ultrastructure in the medium rhabdom with microvillar contributions from seven photoreceptor cells (R1 and R2-R7). R2 to R7 give rise to five radially protruding finger-like structures, with each finger being composed of microvilli from two neighboring receptor cells. The R1 cell produces a large, triangular insertion on one side of the rhabdom. Microvilli are highly aligned in R2-R7, but face in different directions in each cell (enlargement in d). **e** Towards the proximal end of the rhabdom, R1 shows a reduced microvillar contribution, with the remaining six cells forming a symmetrical flower-shaped pattern of six radial fingers. **f** At the proximal limit, the centrally located cytoplasm of the R8 cell becomes visible, with microvillar contributions extending radially from the center. **g, h** Enlargements of e and f, highlighting the regular arrangement of microvilli. **i** Close-up view of the distal tip of the photoreceptor cells protruding into the clear zone, showing desmosomes linking the plasma membrane of adjacent photoreceptor cells (one highlighted by the arrowhead). **j** Schematized representation of the TEM sections at different rhabdom levels, highlighting the overall rhabdom structure and contributions of photoreceptor cells

**Fig. 4:**
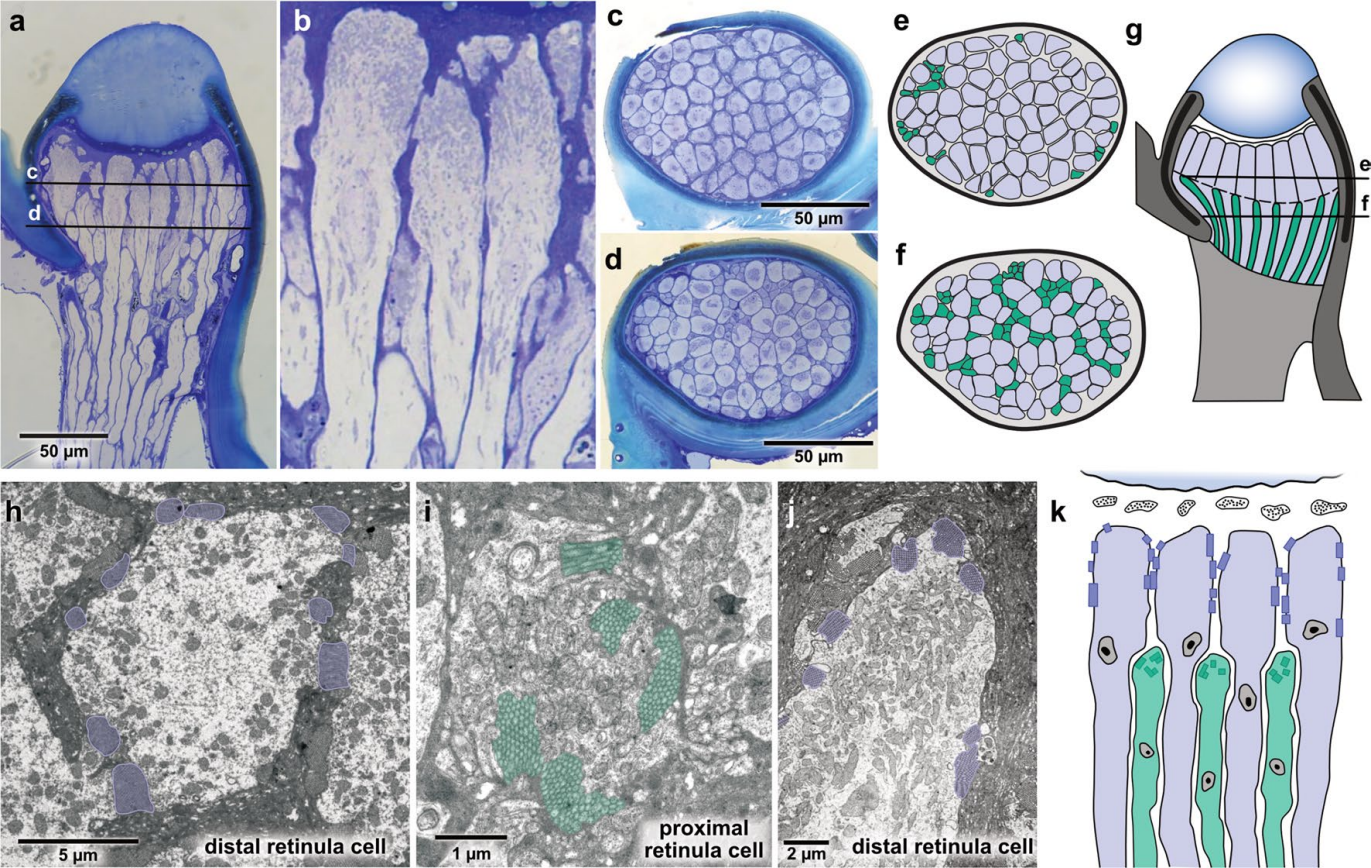
The Bogong moth ocellar retina. **a** Semi-thin longitudinal section of one lateral ocellus showing a two-tiered retina. Levels of sections in c and d are indicated as lines. **b** Enlargement of a, highlighting the large distal and small proximal receptor cell types. **c** Cross section of distal retina with large photoreceptor cells. **d** Cross section of proximal retina with clusters of small photoreceptor cells inserted between the large cells. **e, f** Schematic drawing of cross sections from c and d. Different receptor types highlighted in different colors (purple: large cells; green: small cells). **g** Schematic drawing of ocellus morphology, indicating arrangement of lens, retina and supporting structures. Levels of sections in e and f are indicated. **h** Transmission electron micrograph (TEM) showing a cross section of the distal retina. Large photoreceptor cells have their rhabdomeres (purple overlay) scattered in small patches around each cell. **i** TEM cross section at the level of the proximal retina. The rhabdomeres (green overlay) of the proximal cells lie in the cytoplasm of the cell without direct contact to rhabdomeres of neighboring photoreceptor cells. **j** Longitudinal TEM section of distal photoreceptor cells (purple overlay: rhabdomeres). **k** Schematic drawing of the fine structure of the two tiered retina, based on TEM data

For anatomical analyses of the eyes and ocelli of Bogong moths during their aestivation state (light microscopy, SEM, and TEM of ocelli, Figs. 2, 3, 4), moths were collected from alpine caves during the Australian summer of 2015 and 2016. They were collected in vials, kept at 4$^{\circ }\,\hbox {C}$ for up to one week and then processed for histology at ANU, Canberra. Daylight adapted moths were immobilized by cooling to 4$^{\circ }\,\hbox {C}$. The head capsules were opened to allow fixative to contact brain and ocelli structures directly. The brain was left in the head capsule to prevent distortion and other artefacts, and tissue was fixed in a mixture of 2% paraformaldehyde and 2.5% glutaraldehyde in phosphate buffer (pH 7.2–7.4) for 2–3 h followed by 2% OsO_4_ in distilled water for 1 h. After dehydration and embedding in Araldite (TAAB), semi-thin serial sections of 0.7–1 $\mu$m thickness were cut with a diamond knife (DIATOME). Light microscopy sections were stained with toluidine blue or left unstained, and analysed and photographed with a Leitz DIAPLANE photomicroscope.

### Electron microscopy

#### Transmission electron microscopy

For transmission electron microscopy (TEM) of ocelli (performed at ANU, Canberra) the same moths as for light microscopy were used. Ultrathin sections (40 nm) were cut from the same Araldite embedded samples used for cutting semi-thin sections for light microscopy, using a diamond knife (DIATOME). After collecting these sections on copper grids, they were stained with 6% saturated uranyl acetate (25 min) and lead citrate (5 min) before being imaged with a Tecnai G2 or Hitachi HA7100 electron microscope.

TEM on the compound eyes was performed in Lund, Sweden, on moths collected with light traps during their migration in Kosciuszko National Park during Australian spring (December 2020). Moths were transported to Lund in crowded conditions in plastic containers, and kept in artificial cave conditions (inducing aestivation) for one to four weeks before dissection. Only moths with intact eye glow were used for dissections. Moths were decapitated and heads were fixed in place in a wax dish. After descaling, the head capsule was opened, and the head was submerged in fixative (a mixture of 2% paraformaldehyde and 2.5% glutaraldehyde in 0.1M cacodylate buffer). To facilitate penetration of fixative, the central part of the cornea was removed with a sharp blade. Eyes were then removed from the rest of the head and kept in fixative over night at 4$^{\circ }\,\hbox {C}$. After treatment with 2% OsO_4_ in distilled water for 1 h, the eyes were dehydrated and embedded in Epon resin (Agar 100). Semi-thin sections were cut on an ultramicrotome (Leica Ultracut UCT) using a diamond knife (Diatome) to approach the correct cutting plane. Once an area of interest was reached, several ultrathin sections were cut and collected on copper grids. Grids were then stained with 2% saturated uranyl acetate (30 min) and lead citrate (5 min) and imaged with a JEOL JEM 1400 Plus transmission electron microscope.

#### Scanning electron microscopy

As for TEM studies performed at ANU, scanning electron microscopy (SEM) was performed on the same samples as were used for light microscopy. The heads of the insects were removed with a scalpel and immediately fixed in 2% paraformaldehyde and 2.5% glutaraldehyde in phosphate buffer (pH 7.2–7.4) for 2–4 h. As for TEM preparation, the heads were dehydrated in ethanol and embedded in Araldite (TAAB). After drying, Araldite was carefully pealed off the head cuticle to remove all scales. Finally, the heads were mounted on double sided sticky tape, and sputter coated with gold. We observed the samples either with a ZEISS Ultra Plus or Joel JDM 6400 scanning electron microscope at several different magnifications.

### Micro-computed X-ray tomography

Adult Bogong moths for $\upmu$CT imaging were collected in March 2025, transported to Lund and dissected after three months in aestivating conditions. Moths were decapitated, after which the heads were immediately dipped in 70% ethanol to remove air trapped between scales. The heads were then fixated in 4% paraformaldehyde for 6 days at 4$^{\circ }\,\hbox {C}$ and subsequently transferred to 0.01M PBS. The samples were then incubated in 2% OsO_4_ (in sodium cacodylate buffer) for 48 h at 4$^{\circ }\,\hbox {C}$. Subsequently, the heads were stored in sodium cacodylate buffer at 4$^{\circ }\,\hbox {C}$ until imaging.

To mount the Bogong moth head for imaging, we placed it inside the fine end of a 1 ml pipette tip filled with water, clamped it with a second tip from below and sealed both ends with parafilm to create a stable sample mount.

The moth head was imaged using a Nikon XT H 225 $\upmu$CT scanner equipped with a tungsten transmission target. The scan was performed at 70 kV and 41 $\upmu$A, with a detector gain of 12 dB, exposure of 500 ms with 4 frame averaging, and a total of 4476 projections. The projections obtained were used to reconstruct an image volume using the Inspect-X–3D CT reconstruction software (Nikon) and were saved as a tiff image stack. Cross sections and volume rendering was carried out with either Dragonfly (Dragonfly 2024.1; Comet Technologies Canada Inc., Montreal, Canada) or 3D-Slicer (Fedorov et al. 2012).

### Optical properties of the ocelli

**Fig. 5:**
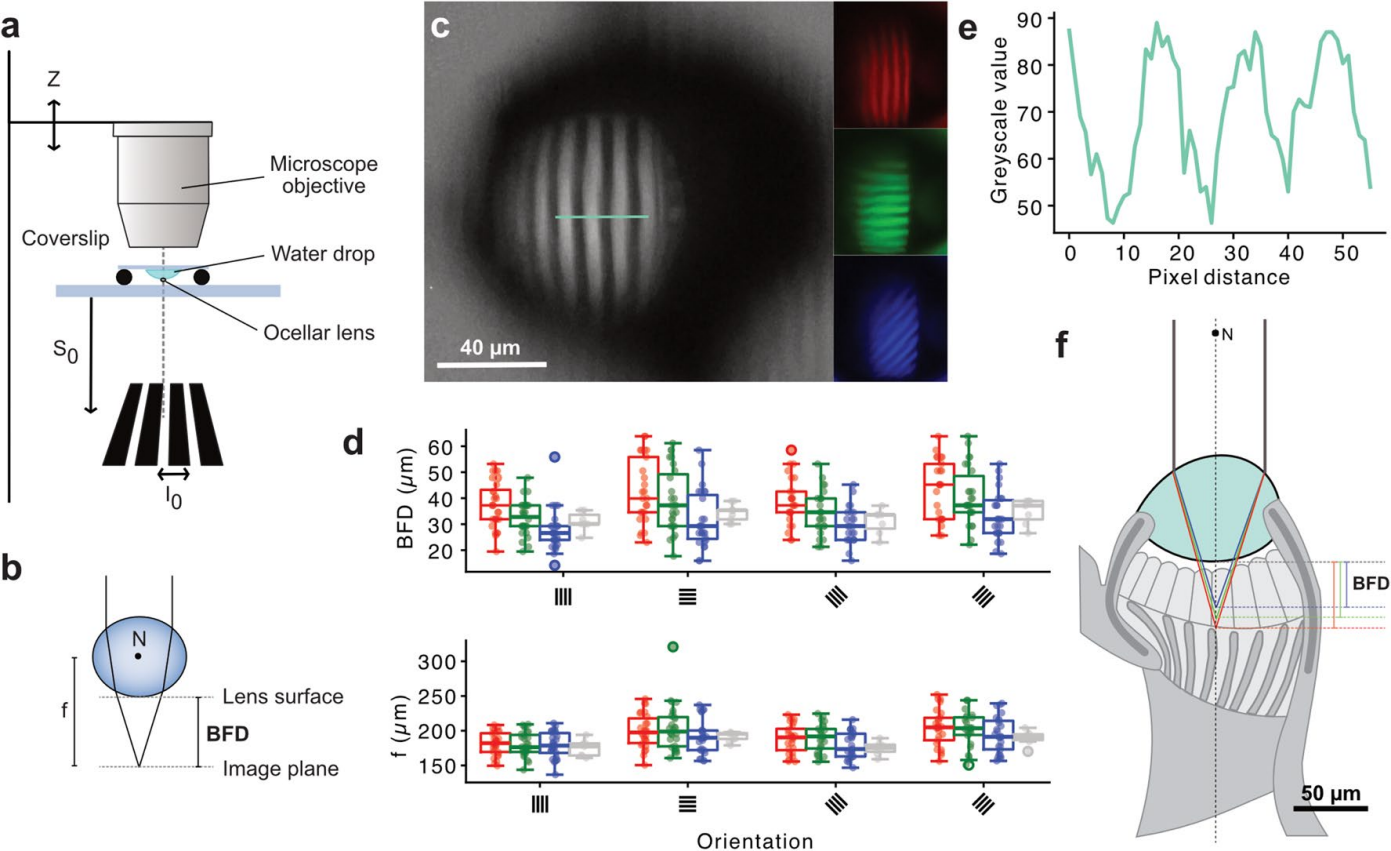
The optical properties of the Bogong moth ocelli. **a** Schematic diagram of the hanging drop method used for analysis of the optical properties of the ocelli. The dashed vertical line indicates the optical axes of the lens and the microscope. **b** Definition of the back focal distance (BFD) and focal length *f*. N is the nodal point. **c** Gratings with various background colors and orientations photographed through the lens of a Bogong moth ocellus. Horizontal blue line indicates the location of the greyscale scan shown in e. **d** Boxplots of the BFD and the focal length *f* as a function of the stimulus orientation (vertical, horizontal, tilted left and tilted right), and colored according to the grating background color. **e** A plot of the greyscale values along the blue line in c. **f** Schematic representation of focal plane location in the Bogong moth ocellus for the three grating colors. Outlines of lens and retina were traced from the ocellus longitudinal section in Fig. 4a

Optical properties of the ocelli were tested in moths collected in November 2019. These individuals were transported to Sweden and kept in an aestivating state in simulated cave conditions. Optical measurements were done using the hanging drop technique described by Homann (1924), carried out identically to Taylor et al. (2016). Briefly, dissected lenses were cleaned in a 0.1M PBS solution, then placed concave side down onto the surface of a distilled water (n = 1.33) droplet itself placed on a glass coverslip. The coverslip was transferred onto a Vaseline-coated-rubber O-ring attached to a glass slide, forming a small evaporation proof chamber containing the downward pointing lens (Fig. 5). The chamber was observed under a Nikon Research Stereo microscope SMZ25/SMZ18.

We placed a screen (Samsung A7 1080*2200 px) under the chamber at a distance of at least 100 mm from the lens (effective infinity). A grating was displayed on an RGB screen via the spacedesk Beta software (datronicsoft, v0.9.45). The grating was generated using a custom-written Python script allowing us to control the color of the stripes, their width and their orientation. We used four distinct grating orientations (0°, 45°, 90°, 135°) to test for lens astigmatism effects, as well as red, blue and green gratings (black stripes on colored background) to test for achromatic effects.

All images were acquired with a Nikon Digital Sight DSu3 camera coupled to a Nikon SMZ18 stereomicroscope and were analysed with the NIS Elements BR 502006 software. We used a p2-SHR plan apo 2x objective (Nikon Metrology GmbH, Düsseldorf) with a numerical aperture of 0.312.

The Back Focal Distance (BFD), that is, the distance between the inner surface of the lens and the plane of best focus, was determined by acquiring an automated Z-stack of images at different planes, and counting the number of frames between the plane of best focus of the grating pattern and the inner surface of the lens. This number was multiplied by the step of the z-stack (for instance 2 $\mu$m) and by the refractive index of the water (1.33).

We measured the focal length *f* using the following formula:

1 $$\begin{aligned} f = s_0 \times \frac{\lambda _i}{\lambda _0} \end{aligned}$$

where $s_0$ is the distance between the grating and the ocellus, $\lambda _i$ the spatial wavelength of the imaged object and $\lambda _0$ is the spatial wavelength of the image of the object.

Back Focal Distances and focal lengths were analysed using a Linear Mixed Model with R (version 4.5.0). We used the R package lme4 to perform the linear mixed models using the grating orientation and background color as fixed effects and the ocellus ID as a random effect.

The quality of the images obtained via the ocellar lens at its focal plane was assessed by determining the Michelson contrast for various gratings whose spatial frequency ranged from 0.1 cycles/deg to 0.5 cycles/deg. We retrieved the greyscale pixel intensity values along a line perpendicular to the grating stripes with a custom-written Python script. The Michelson contrast *M* was computed using the following formula:

2 $$\begin{aligned} M=\frac{(I_{MAX}-I_{MIN})}{(I_{MAX}+I_{MIN})} \end{aligned}$$

where $I_{MAX}$ is the maximal intensity and $I_{MIN}$ is the minimal intensity (i.e. the intensities of the light and dark grating stripes, respectively). The ability to resolve spatial gratings, measured by the Michelson contrast, decreases with increasing spatial frequencies, until it reaches zero (the dark and light stripes are no longer discernible) at a critical optical cut-off frequency $f_c$. The value for $f_c$ was determined by calculating the intersection between a linear regression of the contrast measurements and the x-axis.

### Molecular cloning

Molecular cloning was performed on aestivating moths kept under artificial cave conditions in Lund in 2015. Two aestivating *A. infusa* males were taken from our moth incubator during the night phase. The brain, ocelli and compound eye tissues were dissected and pooled for both individuals. Total RNA was isolated using the RNeasy Mini Kit (Qiagen) followed by removal of genomic DNA with DNaseI (Thermo Scientific). 0.5 $\upmu$g DNase-digested RNA was reverse transcribed with a mixture of oligodT and random hexamer primers using the RevertAid First strand cDNA synthesis kit (Thermo Scientific). 1 $\upmu$l of 1:5 diluted cDNA was used as template for the polymerase chain reaction (PCR). To characterize the opsin repertoire of the Bogong moth, we used oligonucleotide primers based on conserved regions within the untranslated regions (UTRs) of other lepidopteran opsins (based on Xu et al. 2013) (Table S1). For AinfLW2, we used primers targeting a central fragment of the open reading frame (ORF) published for the orthologous opsin from *Agrotis ipsilon* (Xu et al. 2013).

The resulting PCR fragments were cloned into the pTZ57R vector and transformed in competent *E. coli*. Purified plasmid DNAs were verified by Sanger sequencing (3 clones for each opsin from 2 different cDNA libraries). This screen yielded three different types of opsin cDNA sequences encompassing the coding region and extending to the untranslated regions, as well as a partial sequence for AinfLW2. We prepared 5’ and 3’ RACE cDNA libraries and designed opsin-specific primers based on central DNA fragments (Table S1), to amplify the 5’ and 3’ end of the AinfLW2 coding sequence, followed by cloning and Sanger sequencing verification. A pair of gene-specific primers was designed to amplify and confirm the AinfLW2 full-length cDNA sequence, followed by cloning and Sanger sequencing verification.

### Opsin tissue expression

#### RNA sequencing

The state of the Bogong moths used for transcriptome RNA sequencing was carefully controlled. In November 2017, 20 individuals of both sexes (10 females, 10 males) were caught between 20:10-01:04 during their natural southerly spring migration in northern New South Wales, Australia at the Governor Lookout on Mt. Kaputar (elevation 1489 m) using a LepiLED 100 (gunnarbrehm.de) and a white sheet suspended between two trees. Moth heads were individually dropped into ca. 10 ml absolute ethanol immediately after capture. Aestivating moths of both sexes (10 females, 10 males) were collected in a cave on South Ramshead (Kosciuszko National Park) in January 2018. The moths were captured in a plastic container and transported to a rural property near Adaminaby (elevation 1250 m) where they were kept in the fridge to simulate the cave temperature. The next night, between 22:53–01:17, moths were dropped into absolute ethanol, to approximately match the time of day of the migratory moth samples.

Ethanol-preserved moths were used to dissect the antennae, the retinae and the brain. Total RNA from four samples of 5 pooled individuals of mixed sex was extracted using the ISOLATE II RNA mini kit (Bioline) according to the manufacturer’s instructions. The RNA quantity and quality of all samples was determined with a Nanodrop spectrophotometer and assessed on an RNA Nano 6000 Assay Kit of the Agilent Bioanalyzer 2100. mRNA enrichment with oligo(dT) beads, library preparation and illumina paired-end sequencing (HiSeq-PE150) was conducted by Novogene (Hong Kong). Sequence quality, including per base sequence quality and GC content of raw reads, was assessed using FastQC v0.11.3. Reads of poor quality (adapter reads, repeated reads, and reads with low-quality sequencing) were removed using Trimmomatic v0.32 (Bolger et al. 2014) followed by de novo transcriptome assembly using Trinity v3.0 (Haas et al. 2013).

#### RNA extraction retina and ocelli

**Fig. 6:**
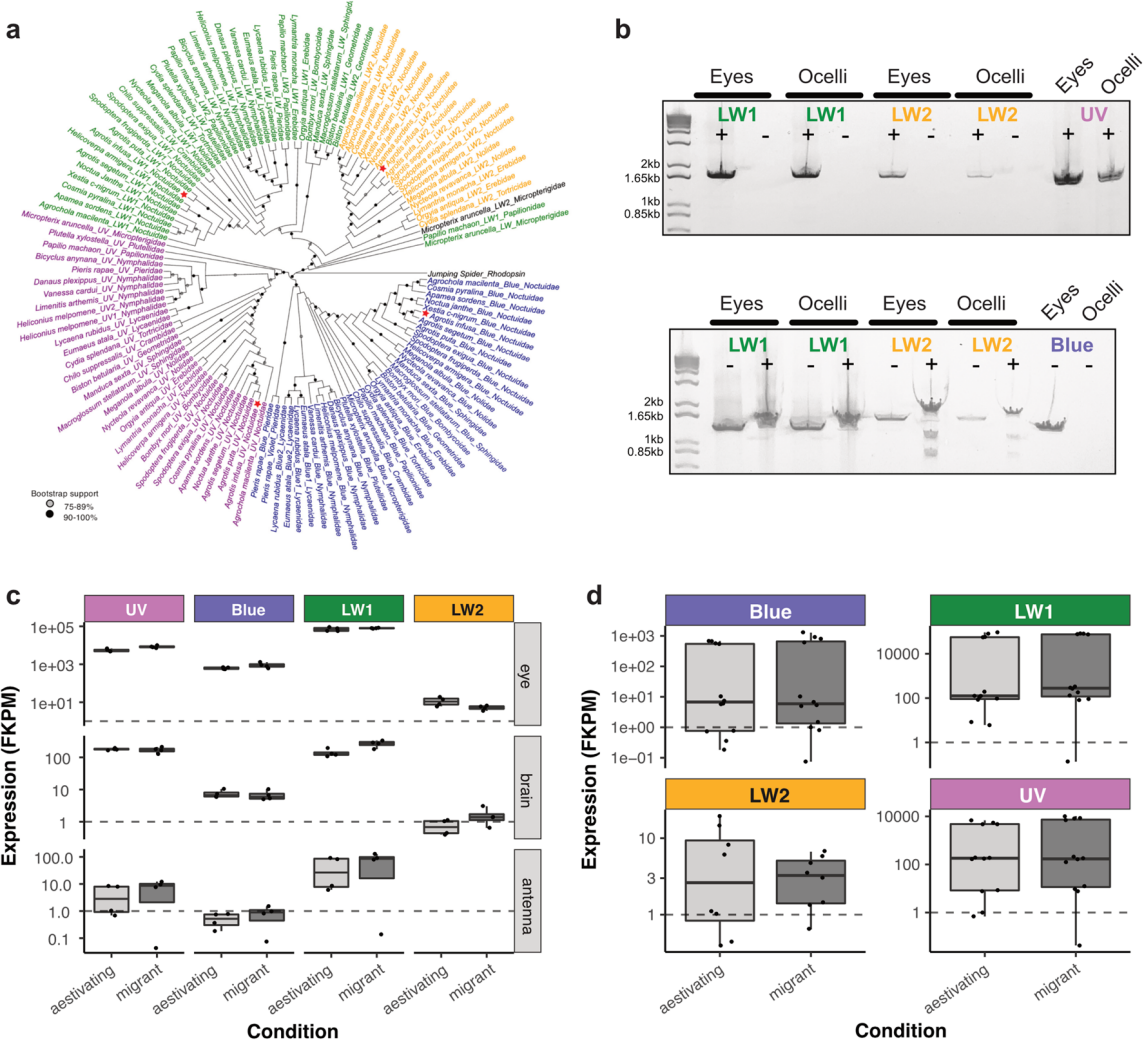
Visual opsins in the Bogong moth. **a** Opsin phylogeny reconstructed from representative lepidopteran species and rooted with the jumping spider rhodopsin. Protein sequences were aligned in MAFFT (Katoh and Standley 2013), and the tree was constructed using IQtree (with 1,000 ultrafast bootstrap, and best-fit model LG+F+I+G4) (Minh et al. 2020), followed by visualisation in Evolview (Zhang et al. 2012; Subramanian et al. 2019). Opsins of the Bogong moth (*Agrotis infusa*) are labeled with a red star. Major opsin clades are colored in purple (UV), Blue (Blue), green (parental LW), and orange (LW retrogene clade of Noctuioidea). **b** PCR expression analysis illustrating that AinfUV, AinfBlue, and both AinfLW1 and AinfLW2 opsins are expressed in the main retina (eyes) whereas AinfBlue is not found in the ocelli. *Top:* PCR amplification with opsin-specific primers from ocellar and compound eye cDNA. ±: cDNA template transcribed with/without reverse transcriptase. *Bottom:* Restriction digest of AinfLW1 and AinfLW2 with *Sac*I. Given the high sequence similarity between AinfLW1 and AinfLW2 (79.9%), and to rule out the possibility of primer cross-annealing, the presence and identity of AinfLW2 was further confirmed by restriction digest specifically cleaving the AinfLW2 amplicon (±: with/without addition of restriction enzyme), establishing that AinfLW1 and AinfLW2 are expressed in compound eyes and ocelli similarly to UV, whereas expression of AinfBlue is restricted to the compound eye retina. First lane, 1kb DNA ladder. Expected amplicon sizes AinfLW1: top: 1736 bp, bottom: 1314 bp, AinfLW2: top: 1707 bp, bottom: 1540 bp (cut by *Sac*I into 638 bp and 906 bp), AinfUV: 1504 bp, AinfBlue: 1306 bp. **c** RNA seq opsin profiling from migrant and aestivating individuals across different tissues. **d** As c, but specifically comparing opsin expression levels in the compound eye retina between aestivating and migrating moths. FKPM, Fragments per Kilobase (of transcript) Per Million mapped reads

10 Bogong moths (5 females, 5 males) were killed in absolute ethanol. Eyes (excluding optic lobes) and ocelli were dissected and pooled. Total RNA was isolated using the Isolate II RNA Micro kit (Bioline) and cDNA was synthesized from 1.4 $\upmu$g of eye RNA and 48 ng of ocelli RNA using the QuantiTect reverse transcription kit with and without (negative control) the addition of reverse transcriptase. 1 $\upmu$l of 1:5 diluted (eyes) or undiluted (ocelli) cDNA was used as template for Touchdown PCR with gene-specific primers (Table S1) and Q5 DNA polymerase (New England Biolabs). Amplicons of expected sizes for the different opsins were visualized by DNA gel electrophoresis. To rule out the possibility that AinfLW2 primers unspecifically amplified AinfLW1 instead of AinfLW2, we cleaned up the PCR reactions with the GeneJET Gel Extraction and DNA Cleanup Micro Kit (Thermo Scientific) and digested with *Sac*I, yielding fragments of defined sizes only in the case of AinfLW2 amplicons (Fig. 6b).

### Opsin functional expression and purification

Coding sequences for Ainf UV and Ainf Blue were flanked by HindIII and BsiWI restriction sites and codon-optimized by Genscript in the pUC57 plasmid. Coding sequences for AinfLW1 and AinfLW2 were amplified by PCR using AinfLW1-pTZ57R and AinfLW2-pTZ57R plasmid DNAs as template and gene-specific oligonucleotide primers (Table S1) flanked by restriction sequences for HindIII and AflII in 5’, respectively, and BsiWI in 3’. AinfUV and Blue plasmid DNAs, and Ainf LW1 and LW2 PCR amplicons were digested and subcloned in the corresponding linear pcDNA5-FLAG-T2A-mRuby2 expression vector (Liénard et al. 2021) followed by cloning using T4 DNA ligase, bacterial transformation in NEB 5-alpha cells (New England Biolabs), and Sanger sequencing verification (Table S1) of all final constructs. Pure endotoxin-free plasmid DNAs bearing each opsin were then transfected in HEK293T cells, and purified under dim-red light. Briefly, for each purification 25 plates were transfected with 24 $\upmu$g pure plasmid DNA in a 2:1 DNA:PEI ratio (40,000 MW, Polysciences GmbH Europe) in Optimem (Gibco). *Cis*-retinal was added 6 h post-transfection, and plates were cultured in the dark for 48 h until cell harvesting and rhodopsin complex formation with 40 $\upmu$M *cis*-retinal. Cell membranes were then solubilized at pH 7.7 using 1% n-Dodecyl-beta-D-Maltoside and opsin proteins in complex with *cis*-retinal were bound to Pierce Anti-DYKDDDDK (FLAG) Affinity Resin (Thermo Scientific) and incubated overnight under gentle rotation prior to purification as described previously in the Pashe procedure validated for LW and SW opsins (Liénard et al. 2021; Roberts et al. 2025). During the purification, the crude protein extract is loaded on a 5 ml Pierce column; non-specific proteins (without FLAG epitope), as well as excess free-retinal, which do not bind to the FLAG resin, are collected in the flow-through fraction and eliminated in successive resin washes. The opsin-FLAG bound fraction is eluted via competitive binding in 1 ml Hepes buffer containing 0.7 mg 3xFLAG peptide (ThermoFisher). The eluate is concentrated to 120 $\upmu$l on an Amicon Ultra-2 10 kDa filtration unit. Dark absorption spectra were measured using a NanoDrop 2000 spectrophotometer (ThermoFisher) or an Implen N60 spectrophotometer (ThermoFisher). For opsins with absorbance in the range 350-450 nm, close to the natural absorbance range of free-retinal, we performed additional assays to confirm protein activity. For the UV opsin, the production of a protonated long-wavelength-shifted photoproduct was monitored via acid denaturation (Tejero et al. 2024). Seven $\upmu$l of protein eluate were gently mixed with 3 $\upmu$l of HCL 133 mM (in water) in a dark 1.5 ml tube, followed by UV–vis measurements. We then obtained a differential spectrum by substracting the dark state spectrum from the acid denaturated spectrum prior to best-fit analysis (Parry et al. 2004). The conversion of *cis*-retinal into retinal oxime, which exhibits maximal absorbance around 360 nm (Parry et al. 2004) was verified for AinfB by mixing a 7-$\upmu$l aliquot of opsin eluate with 0.2 $\upmu$l Hydroxylamine, followed by illumination by bright light for 30 s, then UV–vis measurements.

**Fig. 7:**
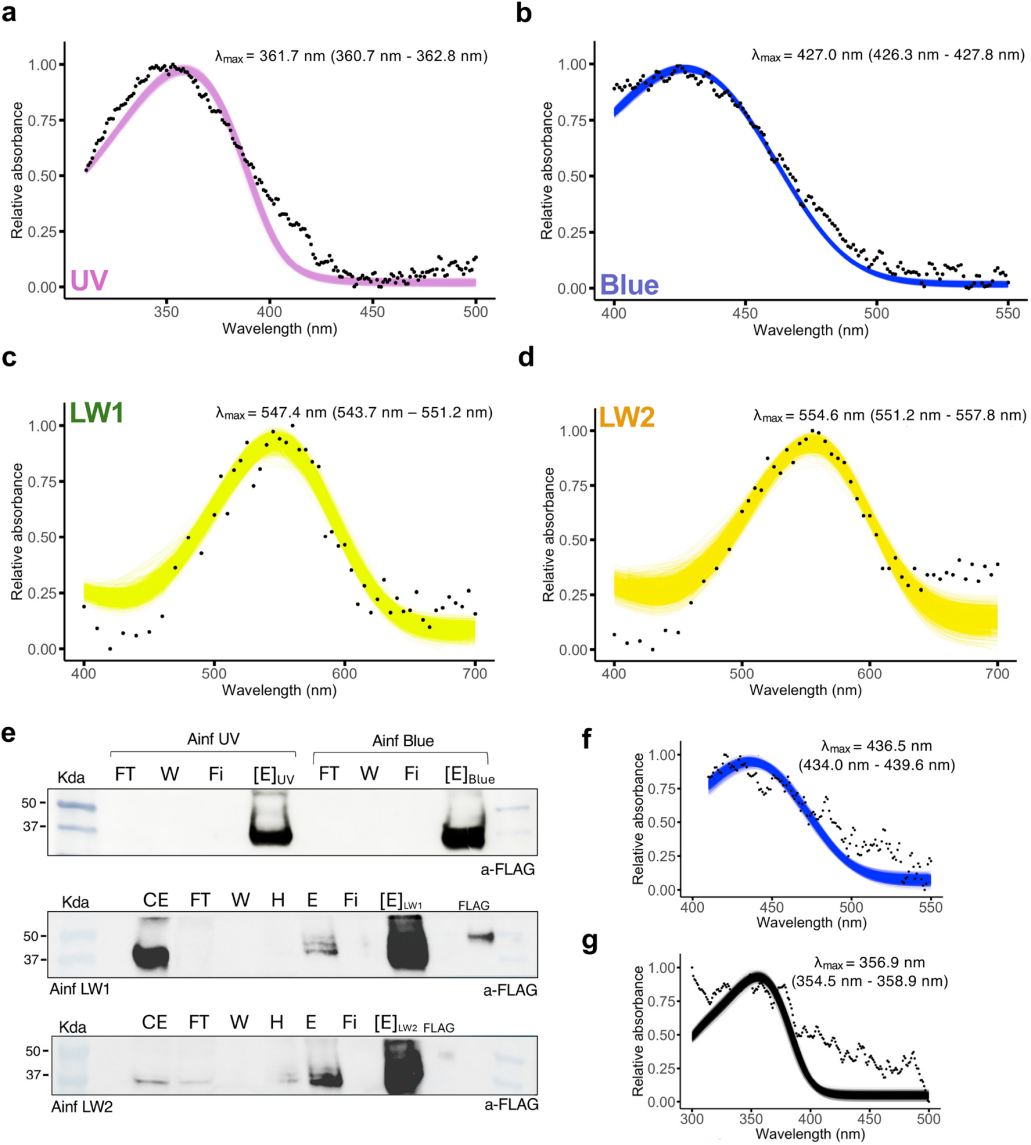
Functional expression of Bogong moth opsins. **a–d** Absorbance curves of visual opsins reconstituted in presence of 11,*cis*-retinal and purified from HEK293T cells. The black dots represent the average absorbance across replicates at every wavelength **a** UV, differential spectrum (dark state n = 2 - acid spectrum), **b** Blue (n = 12), **c** AinfLW1 (n = 9), **d** AinfLW2 (n = 4), where n is the number of measurements of protein aliquots with active opsin complexes. For each opsin, relative absorbance data are fitted using the Govardovskii visual template (Govardovskii et al. 2000) with polynomial function and best-fit analyses computed in R with the colored fitting curves providing estimates of $\lambda _{max}$ following bootstrapping (Liénard et al. 2022). The numbers in parentheses correspond to 95% confidence interval, lower and upper bounds. **e** Western blot analysis of purified opsin fractions. Kda, molecular weight; CE, crude extract; FT, flow through; W, wash; H, water rinse; Fi, filtrate Amicon column; E, unconcentrated eluate; [E], concentrated eluate; FLAG, positive control with pure Flag protein (50 Kda). **f–g** Additional biochemical assays to characterize UV and blue opsins. **f** Acid denaturation assay on AinfUV opsin causes protonation and a blue-shift in absorbance. **g** The addition of hydroxylamine on the purified AinfBlue opsin followed by illumination transforms covalently-bound retinal into retinal oxime with an absorption peak at approximately 360 nm

Equal volumes of purification fractions were mixed with Laemmli buffer supplemented with 10% beta-mercaptoethanol, loaded on a 4–15% Mini-Protean TGX Protein Gel (Bio-Rad) and separated for 100 min at 4$^{\circ }\,\hbox {C}$ and 80 Volts. After transfer to nitrocellulose membranes, blotted bands were blocked using 5% milk in Tris-buffered saline tween 0.5% (TBST) for one hour at room temperature, then incubated overnight at 4$^{\circ }\,\hbox {C}$ with anti-FLAG monoclonal antibody (1:1,000, Sigma Aldrich), washed 3x with TBST, then incubated with HRP-conjugated ECL anti-mouse IgG (1:2,500, GE Healthcare) for 1 h in the dark at room temperature. Signals were revealed using the SuperSignal West Femto (Thermo Scientific) and imaged on an ImageQuant800 (Amersham) (Fig. 7e).

For best-fit analysis, lambda max estimates were calculated by nonlinear least-square fitting to the absorbance data according to the IGOVAR visual template function (Govardovskii et al. 2000) and by performing 1000 bootstrap replication to calculate lambda max predictions and confidence intervals (Liénard et al. 2022) in R v.3.6.6. (R Core Team, 2023) using the packages rsample and tidymodels (Frick et al. 2017; Kuhn and Wickham 2018).

### In situ hybridization

**Fig. 8:**
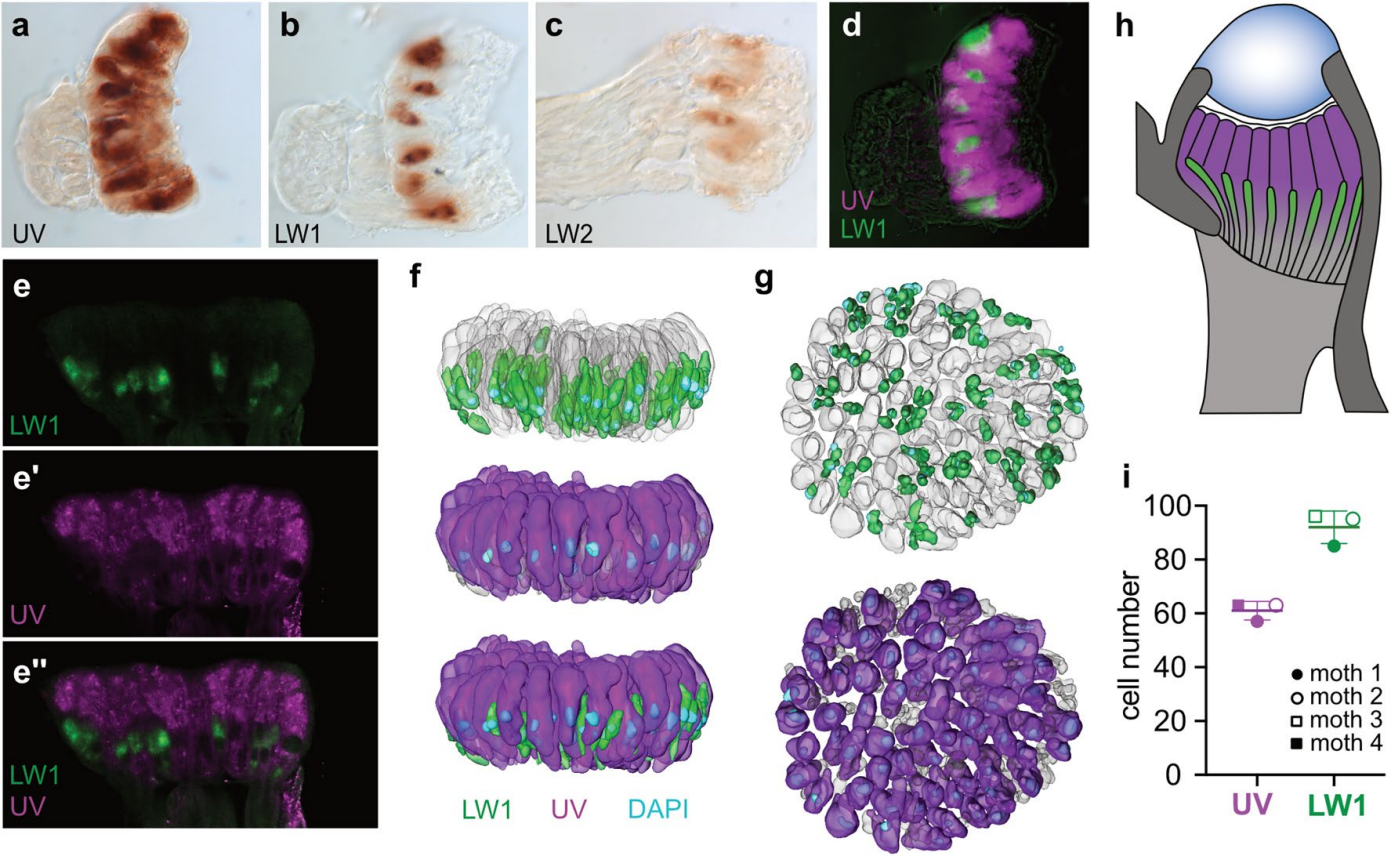
The two layers of the ocellar retina are tuned to capturing specific wavelengths. **a–c** Distribution of spectral classes of photoreceptor cells in the ocellus based on in situ hybridization of cryosectioned ocelli. **d** False-color overlay of LW1 and UV opsin mRNA staining illustrating the expression of both opsins in neighboring photoreceptor cells. **e** Confocal section of whole mount fluorescent in situ hybridisation of UV and LW1 double labeling. **f** 3D reconstruction of ocellar retina, where green and violet colors indicate LW1 expressing and UV expressing photoreceptors respectively; lateral view. Nuclei are stained with DAPI (blue staining). **g** Frontal view of the 3D reconstructed retina shown in **f**. **h** Schematic representation of anatomical layout of green sensitive LW1 and ultraviolet sensitive UV expressing photoreceptor cells in the Bogong moth ocellus. **i** Count of ocellar retina cells expressing either UV or LW1, based on four samples

Bogong moths for in situ hybridization were collected between 2016 and 2018 and used during their aestivation state in Lund for up to six months after capture. An exception was the individual used for Fig. 8e, which was a laboratory raised Bogong moth.

Opsin-specific riboprobes were designed to span parts of coding and UTR regions. The pTZ57R plasmids were used to amplify PCR fragments with the primers listed in Table S1. PCR fragments were cleaned up by gel extraction and subsequently used in *in vitro* transcription reactions with T7- (promoter sequence contained in the pTZ57R plasmid) or SP6- (promoter sequence added with PCR primers) RNA polymerase, using digoxigenin-, biotin-, or fluorescein- labeled nucleotides (labeling mix, Roche). Subsequently, RNA probes were cleaned up using the RNeasy mini kit (Qiagen).

#### DIG-colorimetric in situ hybridization - sections

Moths were removed from the incubator during the dark phase, cooled at 4$^{\circ }\,\hbox {C}$, successively dipped into 70% ethanol and 1x phosphate-buffered saline (1x PBS) and subsequently decapitated.

For eye/ocelli sectioning for in situ hybridization, brains with attached eye and ocelli retinae were dissected in 4% paraformaldehyde in 1x PBS and fixed for 1 h at room temperature. The brains were washed 3x with 1x PBS and incubated in 25% Sucrose in 1x PBS over night at 4$^{\circ }\,\hbox {C}$. Subsequently, they were embedded in Richard-Allan Scientific^TM^ Neg-50^TM^ Frozen Section Medium, frozen and sectioned with a cryostat (Microm HM 560). 14 $\upmu$m thick cryosections were mounted on VWR Superfrost Plus Adhesion slides and stored at -80$^{\circ }\,\hbox {C}$ until use. Slides were thawed, dried for 10 min at room temperature and treated with freshly made acetylation solution (0.1M Triethanolamine + 0.25% Acetic anhydride) shaking for 10 min at room temperature, followed by 3 washes with 1x PBS, and incubation with 2x SSC for 10 min. Slides were prehybridized with hybridization solution (50% deionized formamide, 5x SSC, 50 $\upmu$g/ml Heparin, 250 $\upmu$g/ml torula yeast RNA) for 1 h in a humid chamber at room temperature and subsequently hybridized with 250 $\upmu$l hybridisation solution containing 2 ng/$\upmu$l of riboprobe LW2 and 0.2 ng/$\upmu$l of all other opsin-specific, denatured riboprobes covered with hybrislips at 75$^{\circ }\,\hbox {C}$ overnight in a humid chamber. Following hybridization, slides were placed into 65$^{\circ }\,\hbox {C}$ warm 5x SSC solution to remove the hybrislips. Then, slides were washed 2x 20 min in 0.2x SSC (65$^{\circ }\,\hbox {C}$), 2x 20 min in 2x SSC containing 0.2% Tween 20 at 65$^{\circ }\,\hbox {C}$ and 1x 5 min in 0.1M Tris, 0.15M NaCl (TBS) at room temperature before blocking in 3% blocking reagent (Roche) in TBS, pH 7.5 in a humid chamber at room temperature for at least 3 h. Subsequently, slides were incubated with anti-DIG-alkaline phosphatase-coupled antibody (Roche) at a dilution of 1:5,000 in a humid chamber overnight at 4$^{\circ }\,\hbox {C}$ (500 $\upmu$l/slide). To wash, slides were rinsed 4x 5 min in TBS. They were equilibrated with 0.1M Tris, 0.1M NaCl, pH 9.5 for 5 min before incubation with a NBT/BCIP stock solution (Roche) diluted at 1:50 in the same buffer in the dark. When the staining developed (after 1-3 h), the slides were washed 3x 5 min in 1x PBS containing 0.1% Tween 20 and once more in 1x PBS. They were then embedded in a mixture of PBS:glycerol (1:9) and, after drying, imaged at various magnifications using a light microscope with DIC optics (Zeiss Axiophot).

#### DIG-colorimetric in situ hybridization - wholemounts

For chromogenic wholemount eye in situ hybridization, the eye retinae were dissected in 4% paraformaldehyde in 1x PBS and fixed for 1 h at room temperature in baskets made of cut PCR tubes that were sealed with SpectraMesh (pore size 70 $\upmu$m) and placed in the wells of a 96 well plate. The tissues were transferred to different solutions within these baskets. In situ hybridization was in general performed as described above for the slides with following modifications: For prehybridization and hybridization, tissues inside the baskets were placed into 0.5 ml screw cap tubes, which were put on a Thermomixer shaking at 300 rpm. Prehybridization was done overnight at 75$^{\circ }\,\hbox {C}$ and hybridization was done with 0.2 ng/$\upmu$l for AinfLW1 and 2 ng/$\upmu$l of all other riboprobes at 75$^{\circ }\,\hbox {C}$ over night. Following hybridization, the baskets containing the tissues were washed for 5 min at 300 rpm in 70$^{\circ }\,\hbox {C}$ warm 5x SSC solution. They were subsequently washed at 70$^{\circ }\,\hbox {C}$, 300 rpm, for 2x 20 min in 2x SSC containing 0.2% Tween 20 and 2x 20 min in 0.2x SSC. Then, baskets were transferred to a 96-well plate, washed 1x 5 min in TBS at room temperature and processed for colorimetric reaction as described before for the slides. Eyes were embedded in a 1:9 mixture of PBS:glycerol between two coverslips using 5 hole reinforcement rings as spacers. They were imaged using a Nikon SMZ18 microscope after drying.

#### Fluorescence in situ hybridization on ocelli wholemounts

For fluorescent wholemount ocelli in situ hybridization, moths were dissected as described above and ocelli were fixed over night at 4$^{\circ }\,\hbox {C}$ in 4% PFA in PBS inside PCR-tube baskets as described above. Following fixation, ocelli were washed 3x 10 min in 1x PBS, acetylated in acetylation solution (see above) for 10 min and subsequently washed 3x in PBS and once in 2x SSC. Ocelli were prehybridized in hybridization solution (50% deionized formamide, 5x SSC, 50 $\upmu$g/ml Heparin, 500 $\upmu$g/ml torula yeast RNA, 0.1% Tween 20) in screw cap tubes containing the baskets in a thermomixer at 70$^{\circ }\,\hbox {C}$ shaking at 300 rpm overnight. Hybridization was performed in hybridization solution containing 2 ng/$\upmu$l of AinfUV riboprobe that was labeled with digoxigenin and AinfLW1 riboprobe that was either labeled with biotin (Fig. 8f–i) or fluorescein (Fig. 8e) overnight at 70$^{\circ }\,\hbox {C}$. Tissue was washed post hybridization at 70$^{\circ }\,\hbox {C}$ in a thermomixer once in 5x SSC, twice for 20 min in 2x SSC +0.2% Tween, twice for 20 min in 0.2x SSC + 0.02% Tween and 4x 15 min in 0.1x SSC + 0.03% Triton X-100, then 5 min in TBS. Subsequently, ocelli were blocked in 3% blocking reagent (Roche) in TBS containing 0.03% Triton X-100 over night at 4$^{\circ }\,\hbox {C}$.

Tissues were then incubated with anti-digoxigenin-alkaline phosphatase-coupled antibody (1:500, Roche) and either Streptavidin-Cy5 (1:300, Jackson ImmunoResearch) for detection of biotin-labeled AinfLW1 riboprobe, or anti-fluorescein POD (1:50, Roche) for detection of fluorescein-labeled AinfLW1 riboprobe, in TBS containing 3% blocking reagent and 0.03% Triton for 72 h at 4$^{\circ }\,\hbox {C}$. Ocelli were washed 5x 10min in 0.1M Tris, 0.15M NaCl, pH 7.5, 0.05% Tween 20 (TBS-T) and incubated with 0.1M Tris, 0.1M NaCl, pH 8.0 containing 0.05M MgCl_2_ (DAP buffer) for 2x 10 min. The digoxigenin label was stained by incubation with HNPP1 and Fast Red TR (HNPP fluorescent detection set, Roche) diluted 1:100 in DAP buffer (filtered through 0.2 $\upmu$m nylon filter) in the dark for 2x 30 min at room temperature or at 4$^{\circ }\,\hbox {C}$ overnight. Then, tissues were washed with TBS-T, 3x 5 min. For ocelli stained with a biotin-labeled AinfLW1 probe, samples were washed 1x with PBS and mounted on coverslips using 2 reinforcement rings as spacers in SlowFade Diamond Antifade Mountant containing DAPI (Life Technologies). For ocelli stained with fluorescein-labeled AinfLW1 probe, samples were transferred to amplification diluent containing 1:50 diluted fluorescein-Tyramides (TSA Fluorescein System, Akoya Biosciences) and were stained for 30 min in the dark while shaking. Subsequently, ocelli were washed with TBS-T 3x and once with 1x PBS, then mounted as described above. The ocelli were imaged with a Leica SP7 confocal microscope after drying.

Confocal image stacks were 3D reconstructed with Amira 2020.1 (Thermo Fisher Scientific). Individual retinula cells of the ocellar retina, as well as the DAPI labeled nuclei, were segmented using the Amira segmentation editor. We manually outlined key cross sections in each of the three spatial planes to form a 3D scaffold of each cell. Based on these scaffolds, the full 3D extent of each cell was interpolated using the wrap-tool of Amira. The resulting labelfields were used as the basis for creating a smoothened surface (consisting of triangular faces) for visualization.

## Results

To characterize the visual system of the Bogong moth we examined the compound eyes and the ocelli of this species using a variety of complementary approaches. First, we used light microscopy and micro computed X-ray tomography ($\upmu$CT) to investigate the overall anatomical structure of both eye types, before employing transmission electron microscopy (TEM) to visualize the fine structure of the light detecting structures, the rhabdomes. We then went on to determine the optical properties of the ocellar lenses, to our knowledge, for the first time in any moth. After having established the anatomical basis of Bogong moth vision, we characterized the visual pigments of this species, i.e. the molecular basis of the Bogong moth’s visual abilities. To do this, we cloned the opsin genes, measured their absorption spectra using an *in vitro* expression system and determined their expression patterns in the compound eyes and ocelli via in situ hybridization.

### Morphology of the compound eye

The large compound eyes of the Bogong moth are located on either side of its head and are composed of 3800–4100 equally sized facets, creating a spherical eye surface typical for superposition optics (Fig. 1a–c). No obvious regionalization is visible externally. From the outer surface to the center of the eye, several anatomical zones can be distinguished: the corneal layer, the crystalline cones, the clear zone, the retinula cell layer, the fenestrated layer and the lamina of the optic lobe of the brain (Fig. 2a). The retina and the fenestrated layer are separated by the basement membrane, which forms the boundary between the eye and the brain. The 15 dorsal-most ommatidial rows of the eye form a dorsal rim area (DRA) that is specialized for the analysis of celestial polarized light. Each ommatidium in the DRA possesses a distinct rhabdom structure compared to that found in the main retina (Fig. 2b).

Each ommatidium spans the region from the outer surface of the cornea to the basement membrane (Fig. 2c, d). The acellular, hexagonal corneal lens is transparent, with convex outer and inner lens surfaces (Fig. 2d). Facet diameter varies between 18 and 22 $\upmu$m. The corneal lens can be divided into two layers: a thin epicutical layer (1–3 $\upmu$m) and an inner endocuticle layer (8–10 $\upmu$m thick) (Fig. 2d, f(i)). Beneath the cornea, the cellular components of the ommatidium are composed of three classes of cells: the crystalline cone (or Semper) cells, the pigment cells, and the retinula cells (Fig. 2c–f).

The crystalline cone has a diameter of 14 $\upmu$m and a length of 65 $\upmu$m. It is composed of four cells (Semper cells) (Fig. 2c1, e), in each of which a nucleus is present. The whole body of the cone is enveloped in a transparent thin membrane (1–2 $\upmu$m thick) and connects to the retinula cells at its proximal end. Enveloping the crystalline cone is a one-layered pigment ring generated by two primary pigment cells (Fig. 2c1, f(i)). Secondary pigment cells are elongated cells that stretch between the inner surface of the cornea (distal end) and the basement membrane (proximal end) (Fig. 2c–e). They contain screening pigment granules and are regularly arranged in groups of six cells per ommatidium (Fig. 2c2, d, e, f(ii)). The secondary pigment cells form the clear zone of the eye and the movements of screening pigments provide the means for light–dark adaptation by moving to optically isolate neighboring ommatidia in bright light, or by retracting to allow optical contact in dim light. In bright light, the pigment granules are dispersed within the distal two-thirds of the ommatidium and a few granules are found at its proximal end (Fig. 2f(ii)). Typical for superposition eyes, these pigments migrate distally to aggregate around the crystalline cones in the dark-adapted state, while migrating proximally in the light (not shown). When in the light-adapted state, pigments prevent the rhabdoms of the retinula cells from receiving light refracted from the crystalline cones of neighboring rhabdoms, thus regulating the ability of the eye to collect light. In contrast, in the dark-adapted state, each rhabdom receives light from many crystalline cones, dramatically increasing the effective aperture diameter beyond that of a single ommatidial facet (Warrant 2004).

Each ommatidium contains eight retinula cells extending from the proximal end of the crystalline cone to the basement membrane (Fig. 2c, d). Each of these cells gives rise to a receptor cell axon that passes through the basement membrane to project to the first optic ganglion, the lamina. Within the clear zone, the retinula cells are constricted to a thin thread, which thickens in the proximal part of the ommatidium, where it gives rise to the light-absorbing rhabdomeres, each of which is composed of a stack of microvilli. The eight receptor cell nuclei are located at different levels of the ommatidium: one often in the distal third, six of them in a group thickening the retinula thread in the middle of the ommatidium - occasionally joined by the distal nucleus (Fig. 2c2, d, e), and one at the proximal end of the retina (Fig. 2d, f(iv)). At the level of the rhabdom, the retinula cells are surrounded by a bush of fine tracheoles that form a tracheal sheath that envelopes each rhabdom. These fine tracheoles also form a tapetum (Fig. 2f(iii–iv)). Light entering the ommatidium travels down the rhabdom to the basal membrane, where it is reflected by the tapetum back through the retina to significantly increase light capture. This reflected light creates the familiar eye glow seen in the eyes of moths, including the Bogong moth.

In the proximal portion of the ommatidium, the inner membranes of each retinula cell thicken to form interdigitated microvilli and build a rhabdomere. Together with the rhabdomeres of adjacent retinula cells they build the fused rhabdom. Two different types of rhabdoms can be distinguished. In the 15 rows of ommatidia that constitute the dorsal rim area (DRA), rhabdoms have square-shaped cross sections (Fig. 2b). In the remainder of the eye, rhabdom cross sections are flower-shaped, the morphology typical of non-DRA rhabdoms in other superposition eyes.

As indicated by the different locations of the cell bodies (Fig. 2d), the retinula cells can be divided into three groups. Cells R2-7 form the bulk of the rhabdom. They extend from the distal to the proximal rhabdom, distally overlapping with the R1 cell and proximally meeting the R8 cell (Fig. 3). They form six finger-like processes that extend radially from the center of the rhabdom, each of which is composed of interdigitated microvilli from neighboring retinula cells. In contrast, R1 contributes most microvilli to the distal rhabdom (Fig. 3a–b). At the rhabdome tip, R1 microvilli dominate the rhabdom cross section, but then gradually retreat to one side with increasing rhabdom depth (Fig. 3c–f, j). The R1 rhabdomere intermingles with the finger-like protrusions formed by the R2-R7 cells, forming one of seven overall fingers. Finally, R8, a small basally-located cell, contributes a ring of microvilli to the proximal region of the rhabdom (Fig. 3f, h).

Microvilli of all retinula cells are generally aligned within a single rhabdomere, but point into different angles across the rhabdom. Whether these angles are stable along the length of the rhabdomeres remains to be shown, leaving the possibility of residual polarization sensitivity. In R8, microvilli extend radially from the central cell body (Fig. 3f, h), and in R1, the microvilli appear substantially less ordered compared to the other cells (Fig. 3a). In both cases, any polarization sensitivity can be expected to be eliminated.

Structurally, the retinula cell bodies are stabilized by desmosomes (*Zonulae adhaerentes*), which form tight connections between neighboring cells. These are particularly prominent towards the distal part of the retina (Fig. 3a, i).

### Resolution and sensitivity of the compound eyes

Knowing the anatomy of the Bogong moth’s compound eyes allows us to estimate their anatomical resolution and optical sensitivity. The anatomical resolution is embodied in the interommatidial angle $\Delta \phi$ which, for the spherical optics of a classical superposition eye (like that possessed by Bogong moths), can be calculated from the eye’s radius of curvature *R* and the center-to-center spacing of the facet lenses, which is equivalent to the facet diameter *D*:

3 $$\begin{aligned} \Delta \phi = D/R \qquad (radians) \end{aligned}$$

Since from Fig. 2$D = 22\,\upmu$m and $R = 969\,\upmu$m, this means that $\Delta \phi$ is 1.30°. This is comparable to $\Delta \phi$ in other moths, such as hawkmoths (which are considerably larger and have significantly larger eyes): the elephant hawkmoth *Deilephila elpenor* (1.1°), the hummingbird hawkmoth *Macroglossum stellatarum* (1.2°) and the Tobacco hornworm *Manduca sexta* (0.9°) (Stöckl et al. 2016).

A simple measure of the brightness of an image produced by the optics of an imaging system is given by the system’s F-number, *F* – the lower its value, the brighter the image. The F-number is a unitless quantity defined as the focal length *f* of the optical system divided by the diameter of its aperture (or pupil), *A*:

4 $$\begin{aligned} F = f/A \end{aligned}$$

In the Bogong moth, *A* is the diameter of the superposition aperture of the eye (measured as the diameter of the eye glow – 857 $\upmu$m in Bogong moths: Warrant and McIntyre (1996)). As in all classically built superposition eyes, *f* is the distance from the eye’s center of curvature to the distal tips of the rhabdoms (which is always half the radius of curvature of the eye’s external corneal surface). In Bogong moths, *f* = 485 $\upmu$m (Fig. 2a). Thus *F* = 0.57. This low value indicates that a very bright image is formed on the Bogong moth’s retina, and is comparable to F-numbers found in other nocturnal superposition eyes (Warrant 2008).

The optical sensitivity *S* of an eye is a measure of the fraction of light that the optics of the eye would absorb when viewing a broad-spectrum extended scene of a certain intensity (Kirschfeld 1974; Land et al. 1981; Warrant and Nilsson 1998):

5 $$\begin{aligned} S = \left( \frac{\pi }{4} \right) ^2 A^2 \left( \frac{d}{f} \right) ^2 \left( \frac{kl}{2.3 + kl} \right) \qquad (\upmu m^2sr) \end{aligned}$$

where *d* and *l* are the diameter and length of the photoreceptors, respectively, and *k* is their absorption coefficient (0.0067 $\upmu$m^−1^, a value found in lobster rhabdoms: Bruno et al. (1977)). Wider pupils *A* and shorter focal lengths *f* (i.e. lower F-numbers), or larger photoreceptors, all increase *S*. In Bogong moths, *d* and *l* are respectively 10 $\upmu$m and 94 $\upmu$m (light passes through the rhabdom length twice due to the presence of a reflective tapetum, yielding an effective value for *l* twice that of the physical rhabdom length). Together with the values of *A* and *f* given above, this yields *S* = 41.4 $\upmu$m^2^sr. This is a high value of optical sensitivity that is comparable to the superposition eyes of other nocturnal moths (Cronin et al. 2014, Table 4.1), such as *Deilephila elpenor* (69 $\upmu$m^2^sr), *Macroglossum stellatarum* (38 $\upmu$m^2^sr) and *Ephestia kuhniella* (38 $\upmu$m^2^sr). To put these values into perspective, the value of S typical of a diurnal insect apposition eye would be a few hundred times lower than this (e.g. in the honeybee *Apis mellifera*: *S* = 0.1 $\upmu$m^2^sr), meaning that when viewing exactly the same dim extended scene as the honeybee, the retinae of these moths would absorb hundreds of times more light.

### Morphology of the ocelli

After having found that the morphology of the Bogong moth’s compound eyes is highly similar to that reported for other noctuid moths (Meinecke 1981; Langer et al. 1979), we investigated whether the ocelli showed any specializations. As in other moths, the Bogong moth possesses two ocelli, located behind the antennae on top of the head, just dorsal to the compound eyes (Fig. 1). While surrounded by dense scales, the ocellar lens is clearly visible from the side of the animal, indicating that the ocelli have an unobstructed, lateral field of view, largely towards the horizon, facing slightly forward (Fig. 1c–e). After removing the surrounding scales, the shape of the ocelli became apparent. The entire ocellus is dome shaped, with the shiny, translucent lens resting on top of an otherwise black structure. At the base, this dome is oval with dimensions of 70x90 $\upmu$m (Fig. 1f).

Directly beneath the inner lens surface, a single layer of corneagen cells forms a one-layered epithelium, below which the retinula cells are located. The retina is generally devoid of supporting cells, for instance it contains no tapetum and lacks pigment cells. A few scattered dark-brown pigments are found only at the level of the basement membrane (within the retinula cells) and in the cells enclosing the nerve tract (Fig. 4a). The retinula cells possess axons that pass the basement membrane in a large bundle running towards the dorsal surface of the central brain (Fig. 4a).

### A two-tiered ocellar retina

Despite the small dimensions of the ocelli, our light and electron microscopical examination revealed a complex retinal structure (Fig. 4). Directly beneath the lens, 75 ± 15 (n = 3) large photoreceptor cells of 8-10 $\upmu$m diameter form an uninterrupted, palisade-like layer that spans the complete width of the ocellus. At approximately 50 $\upmu$m depth below the back surface of the lens, a second type of receptor cell intermingles with the large distal cells. These 108 ± 27 (n = 3) proximal receptor cells are smaller, ca. 2–4 $\upmu$m in diameter, and form the proximal retinal layer (Fig. 4a–g).

The two retinal layers also contain rhabdomeres with a distinct structure. In the distal layer of large cells the rhabdomeres are located in between adjacent cells, covering the outer 40–50 $\upmu$m along the photoreceptor length (Fig. 4h, j, k). Microvilli extend from the centrally located cell body of each cell and form patch-like rhadomeres that interdigitate with the microvilli emerging from the adjacent cell. As microvilli protrude radially from all receptor cells, no common alignment of microvillar angles is found. In the small proximal receptor cells, the microvilli are located in internal pockets of the receptor cell bodies, showing a diffuse angular arrangement and covering a depth of around 10 $\upmu$m (Fig. 4i, k).

### Optical properties of the ocelli

Given the elaborate retina of the ocellus, we next asked whether this elaboration was matched by the optics of the ocellar lens or whether, as in most insects, the ocellus is underfocused and unable to provide a sharp image within the retina. Using the hanging drop technique, we projected stripe patterns of defined stripe width through an isolated, down-facing ocellar lens suspended in a drop of water (Fig. 5a). With a microscope, we next focused on the back surface of the lens, from which we focused upwards until the image of the stripe pattern became focused. The distance between this focal plane and the back surface of the lens, when multiplied by the refractive index of the medium (water: 1.33), was defined as the back focal distance (BFD) (Fig. 5b). Using the size of the focused image relative to the size of the object, we then calculated the focal length of the lens (Equation 1). To estimate the chromatic aberration and astigmatism of the lens, the same pattern was projected in different colors (black stripes on red, green or blue background), as well as in four different orientations (Fig. 5c).

Based on measurements from 10 ocellar lenses we found an average BFD of 35 ± 10 $\upmu$m (for all grating colors and orientations). The focal length was approximately 190 ± 24 $\upmu$m (Fig. 5d), yielding a nodal point located far in front of the physical body of the lens. The BFD was largely identical across all grating orientations, although statistical analysis revealed a slightly longer BFD for horizontal (linear mixed model: t = $-$6.756, p$<0.001$) and tilted gratings (linear mixed model: t = $-$5.555, p$<0.001$). For different spectral stimuli, we found a consistent tendency for longer wavelength images being projected slightly further from the lens, with values between 40 ± 11 $\upmu$m for the red grating and 30 ± 9 $\upmu$m for the blue grating, similarly across all grating orientations. This result is consistent with the presence of chromatic aberration in the ocellar lens. While the effect was only significant for the comparison between red and blue grating images (linear mixed model: t = $-$3.072, p = 0.0023), it indicates that shorter wavelengths are generally projected at distances less than 30 $\upmu$m from the lens surface. The image formed would thus fall exactly on the rhabdom regions of the distal retina layer. With focal distances of greater than 30 $\upmu$m, longer wavelength light would form images coinciding with the rhabdom layer of the proximal retina.

While the precise spectral focusing of images into the two retinal layers might be partially blurred by the observed mild astigmatism, our data clearly suggest that the ocelli of the Bogong moth produce a focused image at the level of the retina, an unusual feature for insect ocelli.

To examine if this image is of sufficient quality to resolve features of the visual environment, we projected stripe patterns of varying spatial frequency through the lens (range: from 0.067 cycles/deg to 0.44 cycles/deg). By measuring the Michelson contrast between the dark and bright stripes of each pattern (example in Fig. 5e), we observed that contrast gradually became worse with increasing spatial frequency, and we measured an estimated optical cutoff frequency of 0.501 ± 0.042 cycles/deg (n = 4 ocelli). This value is comparable with that of the much larger ocellar lenses of locusts (Berry et al. 2007b).

### Visual opsin sequences and expression patterns

After characterizing the detailed morphology of the compound eye and ocelli, we next investigated the molecular repertoire of the visual pigments in the Bogong moth and their expression patterns across both eye types.

We characterized four full-length opsin sequences that clustered in distinct established insect opsin phylogenetic clades (Fig. 6a), which were accordingly named AinfUV, AinfBlue, AinfLW1 and AinfLW2. The AinfLW1 and AinfLW2 opsin sequences present 79.9% identity at the nucleotide level and 87.6% conservation at amino acid level. Based on phylogenetic placement, AinfLW2 belongs to a specific clade of retrogene opsins identified within the Noctuoidea (Xu et al. 2016; Mulhair et al. 2023).

To quantify relative opsin expression levels and evaluate potential differential opsin expression between migrant and aestivating adults, pooled cDNA libraries were sequenced to obtain reference transcriptomes of eyes, brain and antenna. Firstly, we observed that opsin mRNA was found in samples from all tissues, but that, as expected, expression levels were consistently several orders of magnitude higher in the eye samples. This indicates that in adults, no opsin has a major role outside of the visual system, e.g. as extraocular deep brain photoreceptor. Second, relative expression levels between the different opsins in the eye samples indicated that AinfLW1 is the most highly expressed opsin, followed by AinfUV and then AinfBlue. AinfLW2 is expressed at comparatively low levels, four magnitudes lower than AinfLW1 (Fig. 6). Finally, both the absolute expression levels, as well as the relative expression ratios, revealed no differences between migrating and aestivating moths. This indicates that despite the dormant state of aestivation, Bogong moths retain a functional visual system.

The amount of mRNA obtainable from the very small ocelli was not sufficient for transcriptome analysis. Thus, to examine the specific expression patterns of the four opsin genes in both ocelli and compound eyes, we performed opsin mRNA expression profiling from cDNA synthesized from compound eye retina or ocelli total RNA. Expression of all opsin mRNAs was detected in the compound eye (Fig. 6b), whereas AinfBlue was not detected in the ocellus. A restriction digest step, using a target sequence only found in AinfLW2, further validated AinfLW2-specific amplification, both in compound eye and ocellus tissues. Consistent with our transcriptome data, the expression level of AinfLW2 was notably lower in all cases.

### Opsin spectral sensitivity

While our phylogenetic analysis predicted sensitivity in the ultraviolet, blue and green range of the visible light spectrum for the four Bogong moth opsins, the precise sensitivity peaks cannot be predicted from sequence data alone and it remains unknown whether the LW2 retrogene opsin may encode a functional opsin, supporting the need to experimentally determine opsin dark absorbance spectra.

The purified opsins, expressed heterologously in HEK293T cells, formed active complexes in solution in the presence of 11,*cis*-retinal (Fig. 7), overall corroborating phylogenetic placement, and with proteins of expected sizes as confirmed via western blot analyses (Fig. 7e). For the *A. infusa* UV opsin, active pigment complexes in the dark absorbed light maximally in the range 350–370 nm (Fig. 7a), with the descending slope of absorption overlapping with the sensitivity of potentially non-covalently bound retinal. To obtain the opsin spectrum, we generated a differential spectrum by subtracting the acid-denatured spectrum from the dark spectrum (Carvalho et al. 2011). In agreement, we observed that the *A. infusa* UV opsin treated in this way indeed yields a photoproduct with maximal absorption at 436 nm (Fig. 7f) validating that AinfUV forms active complexes covalently bound to 11,*cis*-retinal. The difference spectrum was then fitted with the Govardovskii visual-pigment template and returned a best-fit $\lambda _{max}$ of 361.7 nm (Fig. 7a).

The Blue opsin was maximally sensitive at 427 nm (Fig. 7b). Upon light stimulation, the 11,*cis*-retinal is photoisomerized, leading to cleavage of the Schiff base linkage with the opsin Lysine residue. In presence of hydroxylamine, the released retinal reacts to form retinal-oxime, which absorbs in the 355–360 nm range, resulting in a hypsochromic shift of the absorption peak (Fig. 7g) (Parry et al. 2004). Altogether, this confirms that the blue absorbance was due to an active Schiff base in the dark-adapted state, i.e. a functional opsin, equivalent to what can be expected in the eye of the living animal.

The duplicate AinfLW1 and AinfLW2 opsins displayed distinct maximal sensitivity at 547 nm and 555 nm, respectively (Fig. 7c,d), demonstrating an almost 10 nm red-shifted absorption peak in the AinfLW2 opsin.

### Localization of opsins in the ocellar retina

Using the sequence data for the Bogong moth opsins, we designed RNA probes complementary to the mRNA sequence of the moth opsins. These probes were used to detect opsin expression in the ocelli and the compound eyes via in situ hybridization, both in histological sections as well as in whole-mounted preparations.

In the ocelli we were able to detect UV and LW1 mRNA, while no signal was obtained for the blue opsin (Fig. 8a,b,d). We also obtained a weak signal for LW2 mRNA, identical to LW1, indicating co-expression of both long wavelength opsins (Fig. 8c). No signal was found for the no probe or positive strand controls. Similarly, no signal was obtained for the blue opsin, consistent with our mRNA expression profiling data (Fig. 6b). In histological sections, the UV opsin clearly localized to the distal tier of the ocellar retina and was confined to the large receptor cells comprising this layer. The proximal retinal tier, composed of the small receptor cells, was labeled with the LW1 and LW2 probe.

To evaluate the three-dimensional expression profiles of LW1 versus UV mRNA across the two retinal tiers, we additionally performed double fluorescent in situ hybridization of whole-mounted ocelli (Fig. 8e–g). The probes used were coupled to a colorimetric detection system that yielded fluorescence signals of distinct wavelength for either probe. We were therefore able to perform double-labeling experiments, directly observing the relative location of the two signals within the same sample. The results of these experiments further supported the location of the UV opsin exclusively in the large cells of the distal retina layer, with the LW1 opsin being expressed in the small cells of the proximal retinal layer (Fig. 8e–h). As we used DAPI DNA staining to label cellular nuclei (Fig. 8f, g), we were able to obtain precise receptor cell counts. Our initial estimates of cell numbers based on light microscopy were (mean ± range): large cells: 75 ± 15; small cells: 108 ± 27. DAPI DNA staining gave more accurate cell counts (mean ± SD): large cells: 61 ± 3.5, small cells: 92 ± 6.1; Fig. 8i.

In summary, the in situ opsin expression profiles in the ocellar retina confirm cDNA based expression profiling, showing that the distal retinal layer contains UV opsin, while the proximal layer contains a mix of LW opsins. Interestingly, this distribution of spectral sensitivities matches the focal planes of short versus long wavelength light found in our optical measurements, suggesting that the two-tiered retina might have evolved to compensate for the chromatic aberration of the ocellar lens and enable the formation of focused images across a wide spectral range, from UV to orange.

### Localization of opsins in the compound eye

**Fig. 9:**
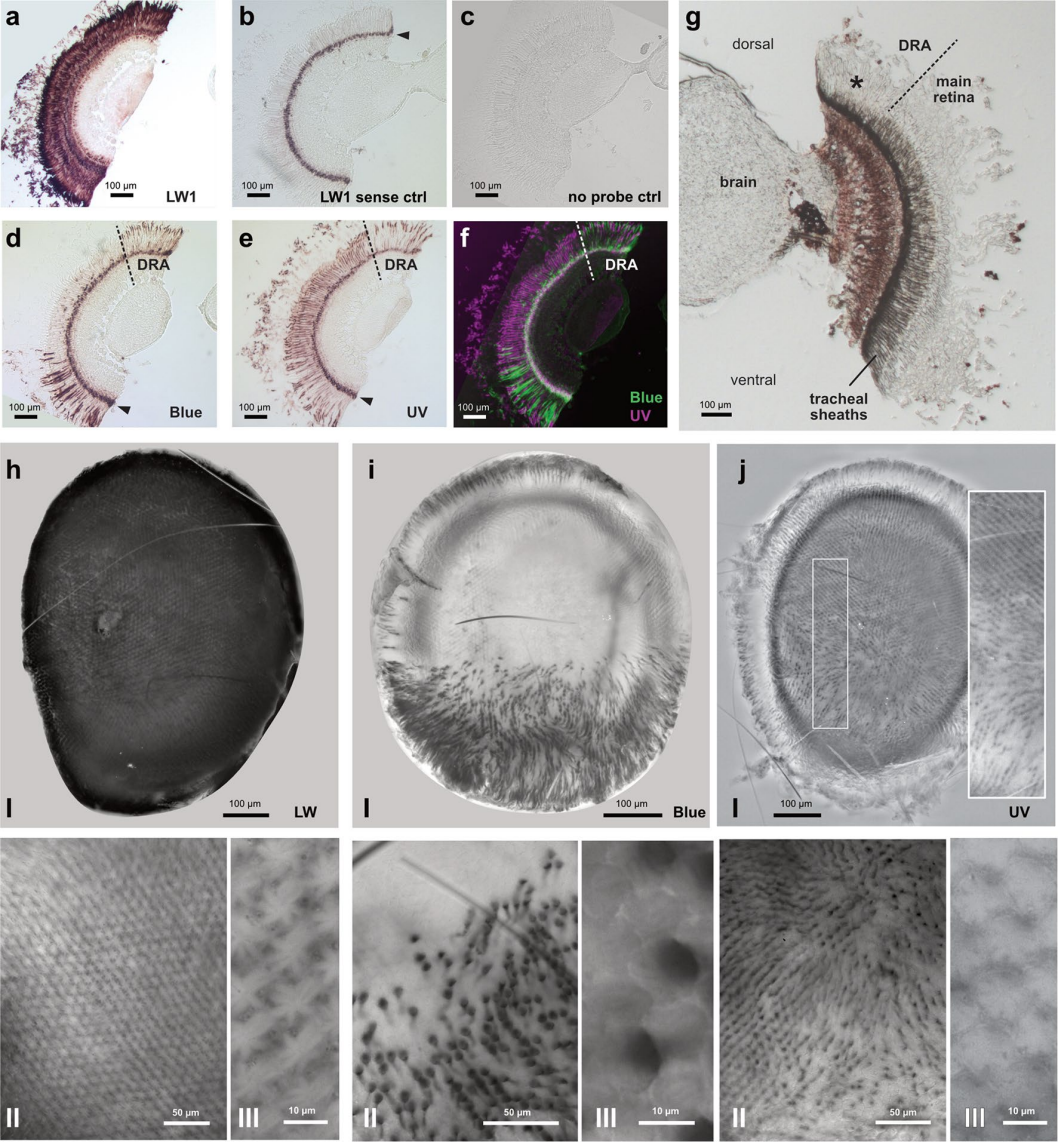
Expression of the three dominant opsins in the retina of the compound eye. **a–c**. Longitudinal microsections of the retina with anti-DIG labeling of long wavelength opsin 1 (LW1) anti-sense cRNA probe (**a**), sense probe (**b**), and no probe (**c**). **d**. anti-DIG labeling with Blue opsin (Blue) cRNA probe. **e**. anti-DIG ultraviolet opsin (UV) expression pattern with cRNA probe. DRA, dorsal rim area. Pointed arrows in b, d and e indicate unspecific background staining. **f**. False color overlay of d and e showing co-localized expression of Blue and UV opsins in the DRA and ventral eye region. **g**. Overview of a microsection of the compound eye comprising the clear zone and main retina, prior to hybridization. The asterisk indicates the DRA. **h–j**. Tangential views of whole mount eye colorimetric in situs with individual LW1 (**h**), Blue (**i**) and UV (**j**) cRNA probes. (II) and (III) panels are zoomed insets from **h**, **i**, **j** panels, respectively. Note that in panels **j** local contrast was equalized to reduce intensity of background staining and aid visibility of receptor staining in the center of the eye

In the compound eye, we were able to localize the three main opsins, LW1, UV opsin and the blue opsin (Fig. 9a–g). The sense controls confirmed the specificity of the probes within the retina, however, a distinct layer near the basement membrane was revealed as unspecific background. As this staining was located in close proximity to the R8 photoreceptor cells, we were not able to resolve the identity of the opsin(s) expressed in this cell.

In our histological sections, the strongest staining was found with the LW1 probe, which labeled the entire retinula cell layer, revealing the rhabdoms as well as numerous cell bodies inside the clear zone (Fig. 9a). In contrast, the blue opsin was expressed in two distinct zones. The dorsally located zone was identified as the part of the retina corresponding to the DRA, whereas the ventral zone was part of the main retina and stretched from just below the eye’s equator to the ventral eye limit (Fig. 9d). Cell bodies within the clear zone were visible, demonstrating that these retinula cells belonged to one of R1-7. The pattern found for UV opsin expression was complementary to that of the blue opsin, with strongest expression in medial regions of the eye, where the blue opsins were not present (Fig. 9e,f). While less abundant in the DRA and across the ventral retina, UV opsin expressing cells were sparsely present in those regions as well. Cell bodies were also located in the clear zone.

To confirm the location of the DRA, we photographed unstained sections prior to in situ hybridization. In those sections, the tracheae enveloping the rhabdoms were visible as a dark layer. In dorsal regions of the eye, this layer was reduced in thickness, limiting the tapetal functions of the tracheal sheaths (Fig. 9g). This is similar to other lepidopterans (*Manduca sexta*, White et al. (2003), Monarch butterfly, Labhart et al. (2009), and dung beetles Yilmaz et al. (2024)), and should reduce self-screening by preventing light being reflected back through the rhabdoms, thus aiding the detection of polarized light.

To confirm the spatial expression zones of the three main opsins in the compound eye, we additionally performed colorimetric in situ hybridization of whole mounted retinae (Fig. 9h–j). These results revealed staining patterns in line with our results with histological sections, and this allowed us to obtain a more precise account of opsin distribution within specific photoreceptor cells (i.e. R1 to R7).

Generally, the opsin mRNA signals localized to the cell bodies of the retinula cells, leaving the finger-like rhabdoms devoid of staining. This pattern resembled the TEM data and showed five evenly spaced rhabdom fingers and one larger protrusion on one side of the ommatidium, presumably corresponding to the R1 cell (Fig. 9h(III)).

LW1 was localized to several cells, consistently leaving a gap on the side of each ommatidium with a large protrusion (Fig. 9h(III)) and thus likely localized to receptor cells R2–R7. In contrast, UV and blue opsins were expressed in one relatively large cell located on one ommatidial side (Fig. 9i(III) and j(III)), supporting UV and blue opsin expression in R1 cells, but not in R2–R7 cells.

## Discussion

In this study we have described the anatomical and molecular layout of the eyes and ocelli of the Bogong moth using a range of complementary methods. We asked whether the ability of the moths to perform highly efficient long-distance migrations would be reflected in specializations in its visual system.

### Compound eye structure of the Bogong moth resembles other noctuid moths

Using several different approaches, our examination of the Bogong moth visual system confirmed earlier findings from other noctuid moths. This is obvious if one considers the overall structure of ommatidia from the Bogong moth’s superposition compound eyes, which is very similar to that found in the African armyworm *Spodoptera exempta* (Meinecke 1981; Langer et al. 1979). The tiered Bogong moth rhabdom closely resembles the layout of that in *Spodoptera*, with eight retinula cells, of which one is a large distal cell (R1), six form the bulk of the rhabdom (R2-R7), and one contributes only to the proximal rhabdom (R8). Finally, a tracheolar network at the basement membrane forms a reflective tapetum, a structure present also in Sphingids and in most butterfly families with apposition eyes except the Papilionidae (Arikawa 2003; Stavenga 2002; Ribi 1979). The tapetum produces the Bogong’s typical eye shine.

Whereas the overall ommatidial layout is similar to that in other studied moth families, such as the Crambidae, e.g. *Ostrinia nubilalis* (Belušič et al. 2017), *Antheraea polyphemus* (Saturniidae) (Anton-Erxleben and Langer 1988) and *Manduca sexta* (Sphingidae) (White et al. 2003), the number of retinula cells and the details of how these cells contribute to the tiered rhabdom varies. In all cases, the rhabdom cross section is flower-shaped, with most of the 8, 9 or more retinula cells contributing to the regular, finger-like protrusions that form the bulk of the rhabdom. While in the ommatidia of the main retina of the Bogong moth only one large retinula cell had microvilli concentrated in the distal rhabdom (R1 cell), in hawkmoths and other investigated moth families, two such cells (called dv-cells) exist in each ommatidium. They are located on opposite sides of the rhabdom and, in some species, are characterized by highly aligned microvilli (Belušič et al. 2017). This alignment equips the dv-cells of those moth species with unusually high polarization sensitivity (Belušič et al. 2017). We did not find similarly ordered microvilli in the R1 cell of Bogong moths, an observation that might warrant further systematic studies to examine the possible sensitivity of the main retina to polarized light. This could be done across adult life stages (and behavioral states). A basal receptor cell contributes to the proximal end of the rhabdom in other moth families as well, generally with a morphology similar to that found in noctuid moths (e.g. Crambidae, Belušič et al. (2017); Saturniidae, Anton-Erxleben and Langer (1988)).

Overall, Bogong moth compound eyes show all of the features previously found in the eyes of noctuid moths. Their eyes also share features in common with more distantly related moth families, for instance in terms of the regionalization of the eye (White et al. 2003; Martín-Gabarrella et al. 2023). We thus conclude that the eyes of Bogong moths do not reveal any obvious specializations for a migratory lifestyle compared to other moths.

### Surprising sophistication of the Bogong moth ocelli

Despite their small size, the ocelli of the Bogong moth possess a complex layout. Their two-tiered retina consists of large, distal UV-sensitive receptor cells, which are complemented proximally by a second receptor cell layer consisting of small cells, co-expressing LW1 and LW2 opsins. Noctuid ocelli have previously been described in another noctuid, the Cabbage looper *Trichoplusia ni* (Dow and Eaton 1976) and our data closely match the reported results from that species. This includes the overall shape of the ocelli as well as their retinal structure.

For the first time in a moth we have determined the focal plane of the ocellar lens. Surprisingly, the image formed by this minute lens was focused within the retina. Our data on chromatic aberration even suggests that long and short wavelength light is focused on appropriate layers of the retina. This is unusual for ocelli, as in many studied insects, ocelli are underfocused (Berry et al. 2007b; Warrant et al. 2006), i.e. the image plane is located behind the retina, producing a blurry image at the retinal level. This implies that the functions associated with the ocelli in these species cannot rely on spatial detail in the images produced, but rather on brightness, spectral information, or the polarization angle. The Bogong moth therefore appears to be one of the few exceptions to this rule, and its ocelli could, at least theoretically, be involved in tasks that require spatial vision.

Underlining this notion, the image produced by the small ocellar lens of the Bogong moth was also found to be of comparably high quality, with limited astigmatism and a spatial frequency cutoff of 0.4 cycles/deg. This value refers to the smallest spatial detail that can still be resolved from a high-contrast stimulus and is comparable to that of much larger ocellar lenses, e.g. from locusts (0.26–0.59 cycles/deg; Berry et al. (2007b)). The projected images also showed no sign of double imaging or multiple focus points, as are produced by the irregularly shaped back surface of the ocellar lenses of locusts and dragonflies.

As ocelli in lepidopteran insects are generally understudied, it is difficult to evaluate how typical the layout of the noctuid ocellus is for moths and butterflies. Several groups of these insects, including hawkmoths, had originally been described as lacking ocelli. This was due to the microscopic size of their external ocelli, which are less than 30 $\upmu$m in diameter (Dickens and Eaton 1973). These external structures are linked to larger internal ocelli, which contain a small number of retinula cells that comprise functional light receptors (Eaton 1971; Dickens and Eaton 1973, 1974; Pappas and Eaton 1977). In butterflies, ocelli are similarly small, but even less well examined (Dickens and Eaton 1973). The only other ocelli anatomically studied in lepidopteran insects, to our knowledge, are those of arctiid moths (family Erebidae) (Grünewald and Wunderer 1996). These largely resemble those of noctuid moths, but possess a non-tiered retina consisting of only a single type of retinula cell. Physiologically, ocellar photoreceptors have been characterized as UV and green sensitive in the European corn borer *Ostrinia nubilalis* (family Crambidae) (Belušič et al. 2017). In no case is it known whether the ocellar lens generates a focused image onto the retina.

Despite the Bogong moth ocellus producing a focused image on the retina, there are several aspects of the ocellar anatomy that nevertheless might prevent the ocelli from detecting a sharp image that provides the brain with spatial information. Firstly, we found no optical isolation of retinula cells by pigments, suggesting that substantial optical cross talk between different receptor cells might occur. Second, the irregular and coarsely spaced rhabdoms generate a sampling array that appears to undersample any high resolution image projected by the lens. Finally, neural pooling is a highly conserved feature across the ocelli of all examined species to date. While neural processing has not yet been studied in the Bogong moth, ocellar photoreceptors generally converge onto only seven large L-neurons and slightly more numerous, and smaller, S-neurons (reviewed in Mizunami 1995; Ribi and Zeil 2017). Thus, even if a sharp image of rich spatial detail is captured at the level of photoreceptors, the downstream neurons would most likely pool this image information into a significantly coarser representation of this input. Yet, these points do not all equally apply to both layers of receptor cells. The small proximal receptor cells are more distantly spaced, and due to their smaller size and close proximity to the focal plane of the lens, cross talk between individual receptor cells is less likely compared to the large distal receptor cells. If neurally, the two layers are processed in dedicated pathways, the proximal retinal pathway could potentially carry spatial information, while the distal pathway would likely not. However, this aspect remains speculative, as no data on the nature of neural processing downstream of the ocellar photoreceptors exists for the Bogong moth. This clearly warrants more research.

Functionally, ocelli have been shown to be involved in flight stabilizing reflexes, both in locusts and dragonflies (Taylor and Krapp 2007; Berry et al. 2006; Simmons and Steveninck 2010). With their lateral and frontal fields of view and sensitivity in the UV and green range of the spectrum, they are perfectly suited to sample the horizon and use the UV-green contrast between the sky and the terrestrial panorama to detect deviations in flight posture. The extremely large downstream neurons (L-neurons) provide a direct and very fast connection to motor circuits in the thorax that control stabilizing reflexes (Simmons 2002).

In bees, ants and wasps, their dorsally facing ocelli likely contribute to the navigational abilities of these insects. This is achieved via detection of polarized skylight (Araújo et al. 2024; Fent and Wehner 1985), a task for which hymenopteran ocelli are suited due to their often highly aligned rhabdomeres (Taylor et al. 2016; Ogawa et al. 2017; Ribi et al. 2011; Narendra and Ribi 2017; Ribi and Zeil 2018). Additionally, wasp ocelli were hypothesized to be involved in the detection of dorsally located landmarks, such as those provided by the tree canopy in dense vegetation (Warrant et al. 2006).

In moths, including the noctuid *Trichoplusia ni*, ocelli have so far been described only as light-level detectors, which contribute to triggering initiation of flight once a specific intensity threshold has been crossed (Eaton et al. 1983; Wunderer and Kramer 1989).

The ocellar functions described above are largely independent of whether the images provided by the ocellar lenses are focused or not. Flight stabilization is possible with underfocused ocelli (e.g. as in locusts), while the focused ocelli of dragonflies are used for the same purpose (Berry et al. 2007a). Similarly, in bees, some ocelli are underfocused, (e.g. in the tropical bee *Megalopta genalis*), while in orchid bees they are focused, which is also the case for wasps (Warrant et al. 2006). The only task that might be dependent on spatial information in ocellar images could be the hypothesized exploitation of dorsal landmark information for navigation in wasps (Warrant et al. 2006). Yet, despite most ocelli providing poor spatial detail, some spatial information is retained in downstream neurons, irrespective of substantial neural pooling (Stange et al. 2002).

In general, ocelli appear to be optimized for maximizing sensitivity and signaling speed at the expense of spatial detail, making them highly suitable for aiding various functions in dim light conditions, including flight stabilization, orientation and navigation, as well as simply gauging the environmental light level.

Which functions might Bogong moth ocelli support? The lateral field of view, combined with their UV-green sensitivity, would make them highly suitable for horizon stabilization during flight, in particular for correcting unwanted roll movements. Another possible function of Bogong moth ocelli is to signal light levels appropriate for initiating flight, a function described in other moths. While polarized light detection is unlikely, given the irregular arrangement of the rhabdomeres, the high quality of the focused ocellar image could possibly aid navigation. The intriguing anatomy and optics of the Bogong moth ocellus clearly calls for future work, including physiological and behavioral experiments to dissect the possible contribution of the ocelli to the behavioral repertoire of the Bogong moth.

### The molecular basis of Bogong moth vision

Both the overall compound eye, as well as the tiered retina show distinct expression patterns of opsin receptors. Overall, we identified four types of opsins in the Bogong moth, including the standard insect repertoire of three opsins tuned to green (LW1), Blue and UV, as well as a second long wavelength opsin (LW2). While all four opsins are expressed in the retina of the compound eye, and encode functional opsins with distinct maximal spectral sensitivities, LW2 was found at much lower expression levels, making its detection in situ unfruitful. However, we precisely mapped the regional and ommatidial expression patterns of all three main opsins across the eye for the first time in a noctuid moth. While LW1 was expressed across all eye regions, as in other lepidopteran eyes studied to date (e.g. Liénard et al. 2021; Frentiu et al. 2015; McCulloch et al. 2016) (but see sexual dimorphism Sison-Mangus et al. 2006), the Blue and UV opsins were located in specific, largely complementary expression zones. Blue expression was limited to the DRA and the ventral eye hemisphere. In contrast, UV was mostly expressed in medial eye regions and was only sparsely present in the remaining eye, a regionalization similar to broader lepidopterans (Arikawa 2003; Briscoe et al. 2003) and other insects such as orthopterans (Henze et al. 2012; Wernet et al. 2015) and hymenopterans (Wakakuwa et al. 2005; Durand et al. 2025). This pattern is also reminiscent of results in the hawkmoth *Manduca sexta*, where Blue opsins also localized to the ventral retina whereas UV opsins were more concentrated in the dorsal eye hemisphere, in particular directly dorsal of the equator (White et al. 2003). In the *Manduca* DRA, a different, yet unidentified, opsin was likely expressed in most cells, while a few cells expressed UV opsin (White et al. 2003). In contrast, the Bogong moth DRA showed all three main retina opsins, with a stronger expression of Blue opsin. These data indicate that regionalization of the compound eye into different zones of spectral sensitivity could be a widespread trait in nocturnal lepidopteran eyes.

In the honeybee, or in lepidopterans equipped with two short-wavelength opsins, distinct photoreceptor cells typically express only one opsin per photoreceptor (but see McCulloch et al. 2022), even if both opsins are found within the same ommatidium. As typically two cells per ommatidium express short-wavelength opsins, these insects possess retinal mosaics of UV-UV, Blue-Blue, or UV-Blue ommatidial classes (White et al. 2003; Briscoe et al. 2003; Arikawa 2003; Spaethe and Briscoe 2005; Wakakuwa et al. 2005). This pattern is generated by a binary ON-OFF cell-fate specification pattern (e.g. Wernet et al. 2015; Rister and Desplan 2011; Perry et al. 2016). The complementary staining pattern from consecutive longitudinal sections in the Bogong moth retina indicates that the Blue and UV opsins are neither co-expressed in the same cells nor in the same ommatidia, but rather localized in one cell per ommatidium. Parsimoniously, this suggests that these opsins are located exclusively in the R1 cell, i.e. the single distal receptor cell. This expression pattern further suggests that ommatidia in the main retina possess one of two possible ommatidial classes, UV-LW1 or Blue-LW1, composed of one UV or Blue photoreceptor alongside six photoreceptor cells expressing the LW1 opsin. This cellular localization is identical to what has been proposed for *Spodoptera* based on selective degradation of R1 microvilli after intense blue and UV illumination of the eye (Langer et al. 1979). Importantly, irrespective of whether one or two distal short-wavelength receptors exist per ommatidium in different species, the proposed mutually exclusive expression of short-wavelength opsins in the Bogong moth could be generated by the same regulatory pathway for cell-fate specification. Nevertheless, additional double fluorescent labeling of tangential ommatidial sections in the Bogong moth would be required to unambiguously confirm this arrangement. If confirmed, the identical receptor layout in *Spodoptera* and the Bogong moth might indicate that this distribution is a general noctuid trait. The possible implications for color vision abilities are discussed below.

The identity of the opsin expressed in the proximal R8 photoreceptor cell could not be definitively resolved due to overlapping background staining. However, data from *Spodoptera* suggests that this cell may express a long-wavelength opsin homologous to LW2, and the low overall expression levels of LW2 in the Bogong moth compound eye would also support its localization to a single photoreceptor per ommatidium.

The LW2 opsin originated via an ancient retroposition event from the parental LW1 opsin in a common ancestor of the Noctuidae, Erebidae and Nolidae moth families. Although retroposition frequently yields non-functional pseudogenes (Jeffs and Ashburner 1991; Zhang et al. 2004), transcriptomic analyses have shown that LW2 is actively expressed in larval heads in species such as *Helicoverpa armigera* (Xu et al. 2016), and *Orgyia antiqua* (Mulhair et al. 2023). In contrast, LW1 has been found expressed in adult heads of both sexes in both species (Xu et al. 2016; Mulhair et al. 2023), suggesting that the two opsins have consistently diverged in stage-specific functions. Our molecular and in situ hybridization results demonstrate that the LW2 retrogene in *A. infusa* is active, albeit with low expression levels. This was the case both in photoreceptors of the retina and the ocelli and suggests a functional role for this opsin. Given the spectral tuning shift towards the orange found for LW2, its expression in the adult could confer a possible evolutionary advantage linked to detecting long-wavelength light in the adult visual system of this species.

### Functional implications for color vision

As we have characterized the spectral classes of opsins and receptor cells, as well as the distribution of these across the Bogong moth’s eyes and ocelli, we can now ask which consequences these characteristics have for the ability of Bogong moths to distinguish colors. The most direct comparison can be made with nocturnal hawkmoths. These moths were shown to possess trichromatic color vision to discriminate colors even in extremely dim light conditions (Kelber et al. 2003a).

**Fig. 10:**
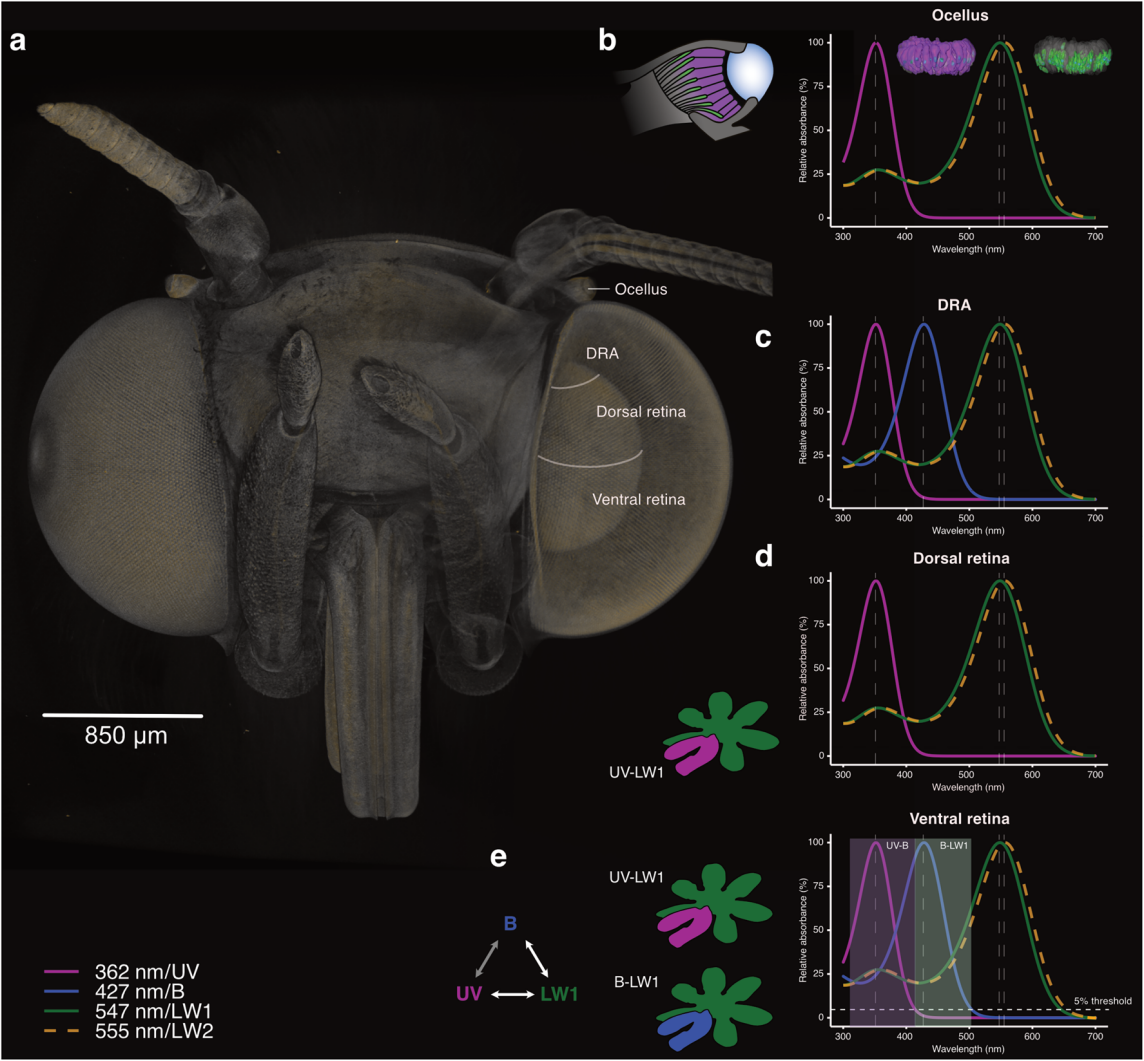
A summary of opsin expression diversity in the visual system of the Bogong moth. **a** A volume rendering of the Bogong moth head (frontal view) based on a $\upmu$CT scan of a female individual. The moth’s left side is shown as semi transparent to visualize the retina beneath the surface of the compound eye. **b** The layout of the ocellus retina with the absorption spectra of the expressed opsins (shown as opsin templates matching the measured peak absorptions of the respective Bogong moth opsins). LW1 and LW2 are coexpressed in the small, proximal photoreceptors. **c** Absorption curves of opsins expressed in the dorsal rim area (DRA) of the compound eye. **d** Absorption curves of opsins expressed in the dorsal retina of the compound eye. Left: Schematic rhabdom cross section generated by UV and LW1 expressing retinula cells. **e** Absorption curves for the ventral and equatorial retina of the compound eye. Different ommatidial types theoretically enable trichromatic color vision by LW1-B, LW1-UV and UV-B opponency. Spectral overlap of the UV-B and LW1-B absorption curves is indicated by the shaded rectangles in the absorption graph. The cut-off for overlap is the calculated 5% sensitivity threshold. Note that a possible LW1-LW2 opponency channel also exists, if LW2 is expressed in R8 receptor cells, as seen in *Spodoptera* (Langer et al. 1979). In the DRA, all three main opsins are expressed, but data on intra- and inter-ommatidial opsin expression patterns is still lacking

Given that the expression patterns of UV and blue opsins differ between the dorsal and ventral hemispheres of the eye, we predict that the ability of Bogong moths to discriminate color also differs markedly between these two hemispheres (Fig. 10).

The dorsal retina of the Bogong moth compound eye largely expresses a single ommatidial configuration of UV-LW1 spectral sensitivity. This configuration presumably yields dichromacy and enables basic color discrimination. For example, most ant species also possess only two opsin genes conferring UV and green sensitivities. However, several species have been shown to discriminate colors when trained with UV and green stimuli (Menzel and Blakers 1976; Yilmaz et al. 2017). This suggests that UV-green opponency processing indeed conveys spectral information rather than providing two parallel brightness channels tuned to different wavelengths. Similarly, dichromatic vertebrate eyes, e.g. in mice (Jacobs et al. 1991), regionally express UV and green photoreceptors and are also able to distinguish UV-green colors (Nadal-Nicolás et al. 2020), although their discrimination ability is less developed than in trichromats.

Thus, it is possible that with its dorsal retina, the Bogong moth can extract not only light intensity information in the UV and green regions of the spectrum, but also, to some extent, relative color information. In contrast to mice or ants however, the spectral distance between the UV-sensitive opsin and the LW receptor is larger in the Bogong moth (LW1 547 nm), compared to the mouse L cone (508 nm), or ant green receptors (ca. 530 nm) (Yilmaz et al. 2017), which may further impact spectral resolution.

In contrast to the dorsal eye hemisphere, the ventral regions offer a richer set of receptor classes. The receptor type B-LW1 alone offers a good overlap in spectral sensitivity ranging from 420 to 500 nm, conferring effective blue-green color discrimination. Since color opponent neurons often compare signals from more than two receptor types (Kelber 2016), adjacent ommatidia expressing UV-LW1 and B-LW1 in the central and ventral retina likely enable UV-B-green trichromacy.

Color vision at long wavelengths ($\ge$500 nm) is in part dependent on spectral overlap and discrimination enabled by comparing Blue and LW1 opsins. Similar to the hawkmoths studied to date, such a mechanism would not allow the Bogong moth to distinguish green-red colors. The extended spectral range of their opsins would nevertheless offer improved achromatic vision by being able to detect a broader wavelength range of light without extracting spectral information. This is particularly valid when compared to diurnal insects such as honeybees, but also applies when compared to nocturnal hawkmoths, whose green opsin receptor cell peaks between 520–530 nm based on ERG data (reviewed in Stöckl and Kelber 2019). The equivalent Bogong moth receptor peaks at ±547 nm. If we consider a visual template fit, and calculate the 5 % sensitivity of the lower absorbance shoulder, this would allow Bogong moths to perceive wavelengths of up to 650 nm compared to approximately 620 nm in e.g. *M. sexta*. Although the relative importance of LW opsins for both chromatic or achromatic functions likely depends on context, additional spectral sensitivity at longer wavelengths has been shown to be beneficial for navigation or feeding behaviours in other insects (Dreyer et al. 2018a; Kelber et al. 2002; Somanathan et al. 2008).

Additionally, the Bogong moth also possesses a second long-wavelength opsin, LW2, which is shifted slightly further into the red range compared to LW1. Supported by complementary evidence in the noctuid *Spodoptera*, we suggest that LW2 is expressed in the basal R8 photoreceptor cell. This opens up the possibility of a receptor channel with an additional spectral sensitivity to distinguish colors associated with long wavelengths in the main retina. Such an ability would require a neural mechanism for direct green (LW1)-red (LW2) opponency. Interestingly, recent evidence from the nymphalid *Charaxes* suggests a similar red-green opponency (Pirih et al. 2022), for which the red channel is generated by a red screening pigment that tunes the single LW green opsin in some receptor cells.

In Bogong moths, the small difference in maximal sensitivity (8 nm) between LW1 and LW2 leads to very high spectral overlap. In an opponent system, this would reduce red-green discrimination compared to other animals with this ability, for instance in humans with M (535 nm) and L (565 nm) cones (Kelber 2016), or papilionid and some lycaenid butterflies, respectively equipped with numerous LW opsins (525, 540, 570 nm) (Arikawa 2003; Saito et al. 2019) or a LW (565 nm) opsin combined with green-shifted blue spectral sensitivity (500 nm) (Sison-Mangus et al. 2006; Liénard et al. 2021). However, basal photoreceptors receive light that is already filtered by more distal photoreceptor cells, possibly yielding an improved signal-to-noise ratio with the potential to increase sensitivity (Pirih et al. 2022). To investigate the possibility of trichromacy involving LW2, a detailed investigation of the photoreceptor projections and their synaptic connections in the lamina would offer an interesting starting point, and potentially reveal convergent evolutionary solutions to similar mechanisms in *Papilio* butterflies.

Overall, the layout of photoreceptor classes in the eyes of the Bogong moth, which form two ommatidial classes (UV-LW and B-LW), suggests that its spectral discrimination ability is likely less advanced than that of hawkmoths or butterflies that are instead equipped with at least 3 ommatidial classes (UV, B, UV-B). The arrangement of photoreceptor classes in Bogong moths is possibly representative of noctuid moths in general. However, only behavioral experiments can ultimately resolve whether these predictions are fulfilled or if the noctuid brain has evolved a novel means of extracting complex spectral information from a simplified receptor layout. Alternatively, the four opsin spectral classes might contribute to achromatic visual functions in ways that support noctuid life history traits.

### Visual specializations that aid migratory behavior?

Given that billions of Bogong moths complete their journey every year, their eyes are clearly suitable to visually guide these animals during their nocturnal migrations. This is reinforced by the fact that these moths have recently been shown to indeed use visual information, in particular the starry night sky, for choosing their migratory bearing (Dreyer et al. 2025). These faint visual cues, in combination with the Earth’s magnetic field, are likely to be the main sensory inputs that guide Bogong moths along their long journey. These sensory demands raise two questions. Firstly, do any characteristics of the Bogong moth’s eyes and ocelli stand out as being particularly advantageous for detecting cues used for migration? Secondly, are any of these features specific to this species, or do they reflect general visual prerequisites for nocturnal migration?

To facilitate their nocturnal migrations, the Bogong moth needs to efficiently detect visual signals in very dim light, including terrestrial as well as celestial cues, both for sensing compass directions and for providing the sensory input that guides stable flight. The compound eyes of Bogong moths are well adapted for this task. The F-number *F* of their eyes is very low (*F* = 0.57), and the image of the dim extended nocturnal world that is focused on the retina will thus be bright. For instance, compare their performance with our own if we both view the same scene. In our own dark-adapted eyes, the F-number is 2.1 (*A* = 8 mm, *f* = 16.7 mm), and since retinal brightness is proportional to 1/$F^2$, this means that image brightness is (1/0.57^2^)/(1/2.1^2^) or 13.6 times brighter on the Bogong moth retina than on our own. This gives the Bogong moth a significant advantage in terms of seeing at night. This difference is also reflected in our respective optical sensitivities (*S* – see Eq. 5): in Bogong moths *S* is 41.4 $\upmu$m^2^sr, while in our own dark-adapted eyes it is only 0.93 $\upmu$m^2^sr (Cronin et al. 2014, Table4.1). This means that the photoreceptors of our eye absorb almost 45 times less light than those of the Bogong moth when we view the same dim scene.

Even though the dim extended world is seen more brightly by Bogong moths than by us, the same cannot be said about its abilities to see the stars, which are tiny points of light. To see more stars, one simply needs a larger pupil. And here we are vastly superior to moths, with a pupil in the dark that is almost 10 times wider than in Bogong moths (8 mm compared to 0.86 mm). Even though we currently do not know how many of the night sky’s brightest stars can be seen by Bogong moths, it will be vastly fewer than the roughly 4,000 we can see (but many more than the 12 stars that can be seen by the apposition compound eyes of the nocturnal shore crab (pupil diameter = facet diameter = 45 $\upmu$m) (Doujak 1985).

Since Bogong moths rely on the stars as a compass for their nocturnal migration (Dreyer et al. 2025), these simple optical calculations suggest that even though the number of individual stars seen by Bogong moths is likely to be limited, the broad extended stripe of the Milky Way is likely to be highly visible. This points to the Milky Way as playing a prominent role for compass navigation in Bogong moths (Foster et al. 2018, 2017; Dreyer et al. 2025).

Compared to the spectrum of light reaching us from the sun and the moon (i.e. sunlight reflected off the lunar surface), starlight contains relatively more red light (Warrant and Johnsen 2013). The absorbance peaks of LW1 and LW2, which are both red-shifted compared to LW pigments in many other nocturnal moths (see above), may thus enhance sensitivity to the longer wavelengths characteristic of starlight.

In the ocelli, LW2 is likely co-expressed with LW1 in the same receptor cells of the ocellar retina. This arrangement likely extends the detectable spectral window of the green-sensitive photoreceptors towards longer wavelengths. The combination of these long-wavelength receptors and the ocellar UV receptors can therefore be expected to be highly suited to detect the contrast between the starlight reflected off the terrestrial panorama and the nocturnal sky, which contains relatively more UV light (Taylor and Krapp 2007; Wilson 1978)

While effective horizon detection during dim light conditions is clearly useful for Bogong moth migration, LW opsins expressed in insect ocelli, such as in odonates, often absorb green-yellow light (Liénard et al. 2022; Roberts et al. 2025) and are also frequently expressed alongside a UV-sensitive opsin (Futahashi et al. 2015; Henze et al. 2012; Chappell and DeVoe 1975). Whereas Bogong moths use the same opsins in the ocelli and the compound eye, in dragonflies and damselflies, green-yellow light is absorbed by distinct ocelli-specific green opsins (Futahashi et al. 2015; Roberts et al. 2025). Similarly, locusts and honeybees also express a distinct LW opsin duplicate in their ocelli (Guignard et al. 2022; Velarde et al. 2005). Like in the Bogong moth, the ocelli of dragonflies and locusts gaze towards the horizon and are known to support flight stabilization (e.g. Stange 1981). Interestingly, both insect groups contain species that perform long-distance migrations. Adjusting the spectral sensitivities of photoreceptors in horizon-facing ocelli might thus optimize the contrast detection between a UV-rich sky and terrestrial long-wavelength cues - whether green foliage in diurnal insects (Möller 2002; Wilson 1978) or reflected starlight in the Bogong moth. This combination of anatomical and molecular ocellar features might serve as a shared, convergent mechanism that migratory insects exploit to enable highly stable flight over extended time periods.

A hallmark of the Bogong moth compound eye was the observed zonation of opsin expression across the compound eye retina, and the associated spectral partitioning. The LW1 opsin was uniformly expressed across the entire eye, in R2-R7 photoreceptors of single ommatidia. This is similar to flies, where it provides the basis for achromatic vision, including movement detection, object detection, and similar tasks. Similarly, the LW1 opsin in the Bogong moth is likely involved in achromatic vision. In contrast, UV and Blue opsins were concentrated in specific eye regions. Blue opsin expression is restricted to the ventral eye hemisphere, while UV opsin expression is found in both hemispheres, although more so in the dorsal hemisphere. This provides one ommatidial class (UV-LW1) in the dorsal eye, and two (UV-LW1, B-LW1) in the equatorial and ventral eye. This distribution is reminiscent of that found in other insects, and closely resembled that of the hawkmoth *Manduca sexta* (White et al. 2003), suggesting functional specialization of both eye hemispheres. In many species, the dorsal eye hemisphere is optimized to view the UV-rich sky, while the ventral hemisphere is better suited to distinguish terrestrial features, characteristics that are useful both for migration and generally for flight in open habitats. Assuming that LW2 is expressed in R8 cells all across the Bogong moth’s retina, the observed 8 nm difference between the peak spectral sensitivities of LW1 and LW2 may either support spectral opponency or enhanced discrimination at long wavelengths (Kelber et al. 2003b). The latter possibility could potentially push the limits of contrast detection in the dim environments illuminated by red-shifted starlight, such as those encountered during nocturnal flight.

In the DRA, all three main opsins are found, with a particularly strong expression of blue opsins. As we were unable to disentangle the distribution of opsins within single DRA ommatidia, it is not possible to infer which receptor is the main DRA receptor. We thus cannot present any functional hypothesis about how these three photoreceptor types are involved in polarized light detection.

Given the limited information available from other migratory and non-migratory lepidopteran insects, it is difficult to draw conclusions about any specific advantages that the described features of the Bogong moth’s visual system may lend to its migratory ability. The general optical and neural strategies that make nocturnal vision more efficient (Warrant 2004) can also be expected to be advantageous for detecting cues relevant for migration. Therefore, the ability to migrate in dim light will ultimately emerge from the moth’s brain, with highly efficient eyes being a necessary prerequisite for evolving visually guided, migratory behavior. In this context, it is interesting to note that while many diurnal butterflies are long-distance migrants, including the iconic nymphalids *Danaus plexippus* (e.g. Reppert et al. 2016) and *Vanessa cardui* (Nesbit et al. 2009), insect-monitoring radar studies in Europe and Asia have observed systematic high mass seasonal emigrations of dozens of noctuid species at high altitudes (200-500 m above ground), e.g. within the genera *Noctua*, *Catacola*, *Agrotis* and *Helicoverpa* (Dreyer et al. 2018a; Wood et al. 2009; Jyothi et al. 2021). While these migrations are in most cases not as targeted as those of the Bogong moth, the sensory challenges are likely comparable. These include visual navigation in dim light, reliable detection of nocturnal celestial cues, and increased need for flight stability. This suggests that the capabilities conferred by the visual system of noctuid moths may be general prerequisites for the evolution of migratory behavior.

## Conclusions

Taken together, this study of the Bogong moth visual system has revealed several interesting features, including sharp ocellar optics and regionalization of spectral sensitivity, both in the ocelli and the compound eyes. Even though these features may not be unique to the Bogong moth, they are an exquisite example of how an insect visual system can evolve highly efficient vision in dim light. While these features are probably typical for noctuid moths in general, the ability to perform long-distance migration is also widespread in this insect group. This indicates that the characteristics of the visual system we have described have likely facilitated the evolution of superior navigational abilities in several different taxa of noctuid moths. The Bogong moth’s ability to perform directed nocturnal journeys of up to 1000 km, to a small set of tiny mountain caves they have never previously visited, is testament to this fact.

## Supplementary Information

Below is the link to the electronic supplementary material.

Supplementary file 1 (pdf 65 KB)

## Data Availability

Spectral measurement data of all opsins as well as microCT data of the Agrotis infusa head have been deposited in the Insect brain database and will be made available upon publication; accession numbers are: EIN-0000279, EIN-0000282, EIN-0000283, EIN-0000284, as well as SIN-0000002 (*Agrotis infusa* species page).
