# Supplementary material for: The visual system of a nocturnal long-distance migrant, the Australian Bogong moth
Source: J Comp Physiol A Neuroethol Sens Neural Behav Physiol. 2025 Dec 18;212(2):359–89. doi: 10.1007/s00359-025-01786-x (PMC13086690; doi:10.1007/s00359-025-01786-x)
Supplement: Supplementary file 1 — (pdf 65 KB) [file 359_2025_1786_MOESM1_ESM.pdf]

Supplementary material

Supplementary tables

Purpose	Sequence 5'-3'
**Amplification from cDNA/opsin characterization**	
AinfLW1-5UTR-s	GGAGTAGTTCGTTTAACGATTTTCTTCAG
AinfLW1-3UTR-as	AAATCGTGTGTCCATAGAGCA
AinfLW2-ORF-s	ATGGCAGACACCGGAC
AinfLW2-ORF-as	CAGCTTGCTGCTTCTCAG
AinfLW2-3RACE-GSP2-s	TGCTATGGCTCCAGCTATGGTTATTAATTGC
AinfLW2-5RACE-GSP1-as	CAGAGGACTAATGGGCGCGCTC
AinfLW2-5UTR-s	CCACATTACGGGCTCACCTT
AinfLW2-3UTR-as	ACAATATTTTAATATGCCGTCCACCG
AinfB-5UTR-s	TCGGACTACTCACTTCG
AinfB-3UTR-as	GCATTGTAGCAGGAGTTG
AinfUV-5UTR-s	CACACTACTGAGGACTGCTC
AinfUV-3UTR-as	ATCTTTTGGAGACAGCATC
**In situ - cRNA probe amplification**	
AinfLW1-probe-SP6-s	ATTTAGGTGACACTATAGAATGATGTGCGCTATGTCTCC
pTZ57R-probe-as	TCACTCATTAGGCACCCCAGG
AinfLW2-probe-SP6-s	ATTTAGGTGACACTATAGATAAGACTGGAGCTCTTCAGC
pTZ57R-probe-as	TCACTCATTAGGCACCCCAGG
AinfUV-probe-SP6-s	ATTTAGGTGACACTATAGAAATATATGATCATGGACATGG
AinfUV-5UTR-as	CACACTACTGAGGACTGCTC
AinfB-probe-SP6-s	ATTTAGGTGACACTATAGATCAACCTGGCAGTCTTCG
pTZ57R-probe-as	TCACTCATTAGGCACCCCAGG
AinfLW1-probe-SP6-s	ATTTAGGTGACACTATAGAATGATGTGCGCTATGTCTCC
AinfLW1-3UTR-as	AAATCGTGTGTCCATAGAGCA
AinfLW2-probe-SP6-s	ATTTAGGTGACACTATAGATAAGACTGGAGCTCTTCAGC
AinfLW2-3UTR-as	ACAATATTTTAATATGCCGTCCACCG
AinfUV-probe-SP6-s	ATTTAGGTGACACTATAGAAATATATGATCATGGACATGG
pTZ57R-probe-as	TCACTCATTAGGCACCCCAGG
AinfB-probe-SP6-s	ATTTAGGTGACACTATAGATCAACCTGGCAGTCTTCG
AinfB-3UTR-as	GCATTGTAGCAGGAGTTG
**RT-PCR**	
AinfLW1-5UTR-s	GGAGTAGTTCGTTTAACGATTTTCTTCAG
AinfLW1-3UTR-as	AAATCGTGTGTCCATAGAGCA
AinfLW1-5UTR-s2	CGGGATTGGTTAAAC
AinfLW1-3UTR-as2	TATGTGATTATTAACCAC
AinfLW2-5UTR-s	CCACATTACGGGCTCACCTT
AinfLW2-3UTR-as	ACAATATTTTAATATGCCGTCCACCG
AinfLW2-5UTR-s2	GAGTAGTCTCCACAGCCTC
AinfLW2-3UTR-as2	TGGTTAAAAACCACCTTG
AinfB-5UTR-s	TCGGACTACTCACTTCG
AinfB-3UTR-as	GCATTGTAGCAGGAGTTG
AinfUV-5UTR-s	CACACTACTGAGGACTGCTC
AinfUV-3UTR-as	ATCTTTTGGAGACAGCATC
**ORF amplification for subcloning in pcDNA5**	
AinfLW1-ORF-HindIII-s	ggtgccAAGCTTGCCACCATGGCGCTGACTCTGGATCCCGG
AinfLW1-ORF-BsiWI-as	cggccgCGTACGGGCGGCAGGTTTCTCCTCGGAAAC
AinfLW2-ORF-AflII-s	ggtgccCTTAAGGCCACCATGGCAGACACCGGACCTGGAAT
AinfLW2-ORF-BsiWI-as	cggccgCGTACGAGCAGCTTGCTGCTTCTCAGACTTC
**pcDNA5 vector primers**	
pcDNA5-s	GCTGTTTTGACCTCCATAGAAGA
pcDNA5-as	TAGAAGGCACAGTCGAGG
pLenti-s	ACCGCATGTTAGCAGACTT
mRuby2-as	CGGCGGCTTAAACCTTATCGTCG

Table S1 Oligonucleotide primers used for cloning, tissue expression PCR, in situ, and heterologous characterization.
Small case letters: extra bases to facilitate restriction digestion; underlined sequences: SP6 polymerase promoter sequence used for riboprobe transcription; UV and BLUE opsins were codon optimized by Genscript, and directly subcloned in pcDNA5, avoiding the PCR amplification step, hence no primers exist for these genes; s, sense; as, antisense.
